# PLZF and its fusion proteins are pomalidomide-dependent CRBN neosubstrates

**DOI:** 10.1038/s42003-021-02801-y

**Published:** 2021-11-11

**Authors:** Nobuyuki Shimizu, Tomoko Asatsuma-Okumura, Junichi Yamamoto, Yuki Yamaguchi, Hiroshi Handa, Takumi Ito

**Affiliations:** 1 Department of Chemical Biology, Tokyo Medical University, 6-1-1, Shinjuku, Shinjuku-ku, Tokyo, 160-8402 Japan; 2 School of Life Science and Technology, Tokyo Institute of Technology, Yokohama, 226-8501 Japan

**Keywords:** Target validation, Ubiquitin ligases

## Abstract

Pomalidomide and lenalidomide are immunomodulatory agents that were derived from thalidomide. Cereblon (CRBN) is a common direct target of thalidomide and related compounds and works as a Cullin Ring 4 E3 ubiquitin ligase (CRL4) with DDB1, CUL4, and ROC1. The substrate specificity of CRL4^CRBN^ is modulated by thalidomide-related compounds. While lenalidomide is approved for the treatment of several diseases including multiple myeloma, 5q- syndrome, mantle cell lymphoma, and follicular lymphoma, pomalidomide is approved only for the treatment of lenalidomide-resistant multiple myeloma. Here we show that PLZF/ZBTB16 and its fusion proteins are pomalidomide-dependent neosubstrates of CRL4^CRBN^. *PLZF* joins to *RARα* or potentially other partner genes, and the translocation causes leukemias, such as acute promyelocytic leukemia and T-cell acute lymphoblastic leukemia. We demonstrate that pomalidomide treatment induces PLZF-RARα degradation, resulting in antiproliferation of leukemic cells expressing PLZF-RARα. This study highlights a potential therapeutic role of pomalidomide as a degrader of leukemogenic fusion proteins.

## Abstract (web summary)

Shimizu et al. identify PLZF/ZBTB16 and its fusion proteins as pomalidomide-dependent neosubstrates of the cereblon E3 ligase. The authors also report that pomalidomide induces their degradation and thereby exerts its anti-cancer effect in leukemic cells expressing the fusion proteins.

## Introduction

Pomalidomide and lenalidomide are thalidomide derivatives developed as immunomodulatory compounds in the mid-1990s^1^. Pomalidomide has an additional amino group attached to a phthaloyl ring of thalidomide, whereas lenalidomide lacks one carbonyl group of pomalidomide. These compounds have been shown to be up to 50,000 times more potent at inhibiting TNF-α production than the parental compound, with pomalidomide being around 10 times more potent than lenalidomide^2^. These drugs have been shown to possess potent anti-myeloma activity. Multiple myeloma is a B-cell malignancy and known as a refractory disease. Pomalidomide and lenalidomide are effective in the treatment of multiple myeloma and other types of blood cancer^3,4^.

Pomalidomide, lenalidomide, and thalidomide have a common primary target, CRBN, a substrate receptor of CRL4 E3 ligase that is complexed with DDB1, Cul4, and Roc1^5^. CRBN is a 441- or 442-amino-acid ubiquitously expressed protein that is evolutionally conserved from plants to humans^5^. When a thalidomide-related compound binds to CRBN, neosubstrates are recruited to CRBN and ubiquitinated for proteasomal degradation^6^. Various drug-dependent neosubstrates are recruited to CRBN in a cell type- and/or compound-specific manners. In multiple myeloma cells, pomalidomide and lenalidomide induce the breakdown of Ikaros (IKZF1) and Aiolos (IKZF3), resulting in IRF4 downregulation and cell growth defects^7–9^. Thalidomide also degrades Ikaros and Aiolos, although less effective^10^. Pomalidomide also induces degradation of ARID2, a subunit of the chromatin remodeling complex PBAF and thereby exerts anti-myeloma effect^11^. In developing embryos, thalidomide induces the degradation of ΔNp63α and TAp63α, gene products of *TP63*, and thereby causes limb and ear defects, respectively^12^. In embryonic stem (ES) cells and induced pluripotent stem (iPS) cells, thalidomide, pomalidomide, and lenalidomide induce the degradation of SALL4, which might also be involved in thalidomide teratogenicity^13–15^. In myelodysplastic syndrome 5q- cells, lenalidomide, but not pomalidomide or thalidomide, induces degradation of casein kinase 1α (CK1α), a critical regulator of 5q- cell growth, through CRL4^CRBN10^. In acute myeloid leukemia cells, CC-885, but no other CRBN-binding drugs, drives the breakdown of the translation termination factor GSPT1, resulting in antiproliferation^16^. Several 3D structures of CRBN-drug-neosubstrate complexes have been determined^16–19^. CRBN-binding drugs work as molecular glues^20^ connecting CRBN with neosubstrates; different neosubstrates can be recruited by different drugs, particularly due to the difference in phthaloyl moiety. For example, the lack of a carbonyl group of lenalidomide avoids steric clash, allowing for binding of CRBN to CK1α^17^. The extended chloro-methyl phenyl group and urea structure of CC-885 contribute to the interaction between CRBN and GSPT1^16^. The amino group of lenalidomide and pomalidomide binds to a critical glutamine residue of Ikaros and Aiolos and enhances their interactions with CRBN^18^. Most of the neosubstrates identified so far have at least one β-hairpin motif containing a specific glycine that is critical for their interactions with CRBN^16–18^.

Among approved CRBN-binding drugs, lenalidomide is currently widely used for not only multiple myeloma but also MDS 5q- syndrome^21^, mantle cell lymphoma^22^, and follicular lymphoma^23^. However, pomalidomide is limited to relapsed or refractory multiple myeloma^24^. To expand the clinical use of pomalidomide, we investigated “neosubstrate-based” drug repositioning, which employs the function of neosubstrates to consider therapeutic applications of the drug. We screened pomalidomide-specific neosubstrates associated with diseases from various tissues to find out the therapeutic effects of pomalidomide.

Here we show that zinc finger and BTB domain containing 16 (ZBTB16)/promyelocytic leukemia zinc finger (PLZF) is a pomalidomide-specific neosubstrate. *PLZF* undergoes chromosomal translocations to cause leukemias such as acute promyelocytic leukemia (APL) and T-cell acute lymphoblastic leukemia (T-ALL). A fusion protein composed of PLZF and Retinoic Acid Receptor A (RARα) is associated with a rare type of APL and has been well characterized^25^. Moreover, a fusion gene comprising *PLZF* and *ABL1* is found in some patients with T-ALL^26^. We demonstrate that pomalidomide drives proteasomal degradation of PLZF-RARα through CRL4^CRBN^ at pharmacologically relevant concentrations, resulting in antiproliferation of leukemic cells. Moreover, we show that PLZF-ABL1 is also downregulated by pomalidomide. This study highlights a therapeutic role of pomalidomide in certain types of leukemia.

## Results

### PLZF is a pomalidomide-dependent CRBN-binding protein

We first sought to identify pomalidomide-dependent CRBN-binding proteins from various cell extracts. The CRL4^CRBN^ complex was purified using anti-FLAG agarose from 293T cells stably expressing FLAG-HA (FH)-CRBN. Then, purified CRL4^CRBN^ was incubated with another cell extract in the presence or absence of pomalidomide for coimmunoprecipitation using anti-HA agarose beads. From long-term neuroepithelial stem (lt-NES) cell extracts, several pomalidomide-dependent CRBN-binding proteins were identified by LC/MS, such as ZBTB16/PLZF, methionine-tRNA ligase, guanosine monophosphate synthetase, and several metabolic enzymes (Supplementary Fig. 1a–c and Supplementary Data 1). PLZF is a 673-amino-acid, C2H2-type zinc finger (ZF)-containing transcription factor. Since many of the known neosubstrates of CRL4^CRBN^ contain a β-hairpin motif flanked by a C2H2-type ZF motif^18,27^, we hypothesized that PLZF would serve as a neosubstrate of CRL4^CRBN^.

**Fig. 1:**
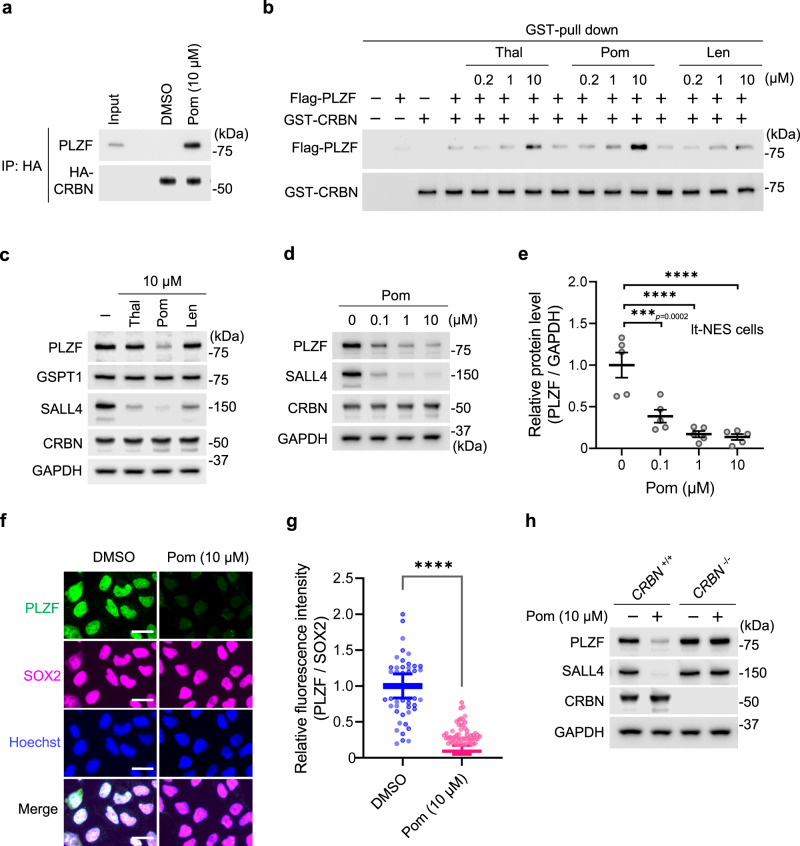
PLZF is a downstream target of the CRBN-pomalidomide pathway. **a** The CRL4^CRBN^ complex containing FH-CRBN was incubated with lt-NES extracts and then subjected to immunoblotting with the indicated antibodies. **b** The interaction between purified recombinant GST-CRBN and PLZF in the presence of the indicated compounds was analyzed by immunoblotting. **c**, **d** Lt-NES cells were incubated with DMSO or one of the indicated compounds at the indicated concentrations for 24 h and harvested for immunoblot analysis. **e** The immunoblots shown in **d** were quantified, and relative intensities of PLZF/GAPDH were calculated and normalized to the control value (DMSO). Data are shown as mean± SEM from five biologically independent samples from two independent experiments. ***p < 0.001, ****p < 0.0001, one-way ANOVA with multiple comparisons test. **f** Immunofluorescence staining of PLZF (green), SOX2 (magenta), and Hoechst (blue) in lt-NES cells treated with DMSO or 10 µM pomalidomide. Merge represents the stacked images of PLZF, SOX2, and Hoechst. Scale bar, 20 µm. **g** From the immunofluorescence data in **f**, relative intensities of PLZF/SOX2 were calculated and normalized to the control value (DMSO). Data are shown as mean ± SEM from three biologically independent samples (*n* = 434 for DMSO and *n* = 405 for 10 μM pomalidomide treatment). ****p < 0.0001, two-sided Mann–Whitney *U*-test. **h** The PLZF protein levels in parental and *CRBN*^−/−^ lt-NES cells were analyzed by immunoblotting. All experiments were conducted more than twice independently, and representative data sets are shown.

To confirm the pomalidomide-dependent interaction between CRBN and PLZF, Myc-FLAG (MF)-tagged PLZF was ectopically expressed in 293T expressing FH-CRBN. Immunoprecipitation using anti-HA antibody showed that CRBN binds to PLZF only in the presence of pomalidomide (Fig. 1a). We confirmed this interaction by coimmunoprecipitating endogenous CRBN and PLZF from lt-NES cell extracts (Supplementary Fig. 2a). In vitro binding assay using GST-fused CRBN and FLAG-tagged PLZF also confirmed pomalidomide-dependent binding of CRBN to PLZF (Fig. 1b and Supplementary Fig. 2b). The interaction was increased by pomalidomide in a dose-dependent manner, but not by thalidomide or lenalidomide substantially (Fig. 1b).

### PLZF is a pomalidomide-dependent neosubstrate of CRBN

We next investigated whether pomalidomide affects the PLZF protein level at pharmacologically relevant concentrations. Lt-NES cells were treated with pomalidomide, lenalidomide, or thalidomide at 10 μM for 24 h (Fig. 1c). Pomalidomide, but not thalidomide or lenalidomide, markedly decreased the PLZF protein level, while all the compounds decreased SALL4, a previously reported neosubstrate (Fig. 1c). Time-course analysis showed that pomalidomide substantially decreases the PLZF protein level in 3 h (Supplementary Fig. 2c, d). The peak plasma concentrations of these compounds are ~5.4 μM (thalidomide), ~2 μM (lenalidomide), and 0.2 μM (pomalidomide)^28^. Concordantly, pomalidomide decreased the PLZF protein level by more than 50% at 0.1 μM (Fig. 1d, e), suggesting that pomalidomide affects PLZF expression in vivo. Immunofluorescence analysis confirmed the decrease of PLZF by pomalidomide in living cells (Fig. 1f, g). The decrease of PLZF by pomalidomide was confirmed by using other cell lines such as human neural stem cell line Sai2 and human acute myeloid leukemia cell line KG1 (Supplementary Fig. 2e–g). By using genome editing, we established lt-NES cells lacking the *CRBN* gene. Pomalidomide did not affect the PLZF protein level in CRBN-deficient cells (Fig. 1h), demonstrating that CRBN is required for pomalidomide-induced downregulation of PLZF.

**Fig. 2:**
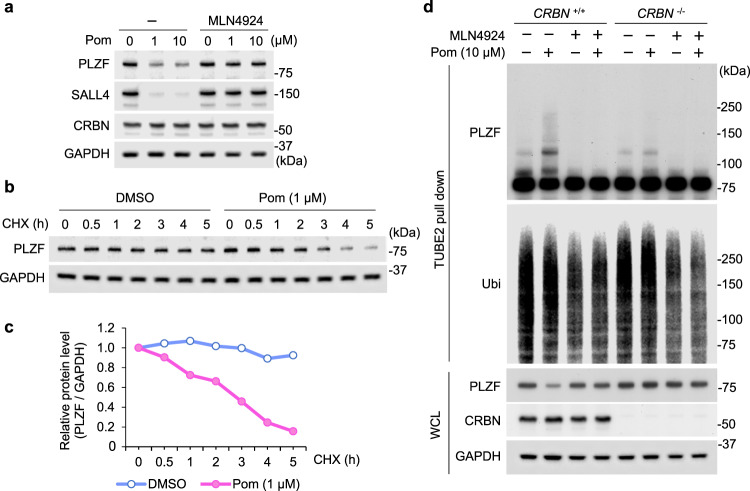
PLZF is a pomalidomide-specific neosubstrate of CRL4^CRBN^. **a** Lt-NES cells were incubated with DMSO or pomalidomide at the indicated concentration for 6 h and harvested for immunoblot analysis. Cells were pretreated with DMSO or 1 μM MLN4924. **b** Immunoblot analysis of lt-NES cells treated with 100 µg/mL CHX and DMSO or 1 μM pomalidomide for the indicated periods. **c** PLZF protein levels in **b** were quantified, and relative intensities of PLZF/GAPDH are shown. **d** Wild-type or *CRBN*-deficient lt-NES cells were treated with 10 µM pomalidomide and/or 1 µM MLN4924 for 7 h and subjected to affinity purification of ubiquitinated proteins using TUBE2 agarose beads. WCL whole-cell lysate. All experiments were conducted more than three times with similar results.

To investigate whether PLZF is a neosubstrate of CRL4^CRBN^, lt-NES cells were co-treated with pomalidomide and MLN4924, a neddylation inhibitor. As expected, the decrease of PLZF was reversed by MLN4924 (Fig. 2a), pointing to the involvement of a Cullin-based E3 ligase. In addition, cycloheximide treatment showed that the protein half-life of PLZF was shortened by pomalidomide (Fig. 2b, c and Supplementary Fig. 3a, b) whereas its mRNA level was unaffected by pomalidomide (Supplementary Fig. 3c). Furthermore, the cellular ubiquitination status of PLZF was examined in the presence or absence of CRBN-binding drugs. PLZF ubiquitination was induced by pomalidomide, but not by thalidomide or lenalidomide substantially (Fig. 2d and Supplementary Fig. 3d). Under the same conditions, SALL4 ubiquitination was induced by all the CRBN-binding drugs tested (Supplementary Fig. 3d). In CRBN-deficient cells, pomalidomide-induced ubiquitination of PLZF was not observed (Fig. 2d). Lastly, in vitro ubiquitination assay showed that PLZF is directly polyubiquitinated by CRL4^CRBN^ in the presence of corresponding E1 and E2 enzymes and ATP in a pomalidomide-inducible manner (Supplementary Fig. 3e). These results led us to conclude that PLZF is a pomalidomide-dependent neosubstrate of CRL4^CRBN^.

### Identification of two structural degrons in PLZF

**Fig. 3:**
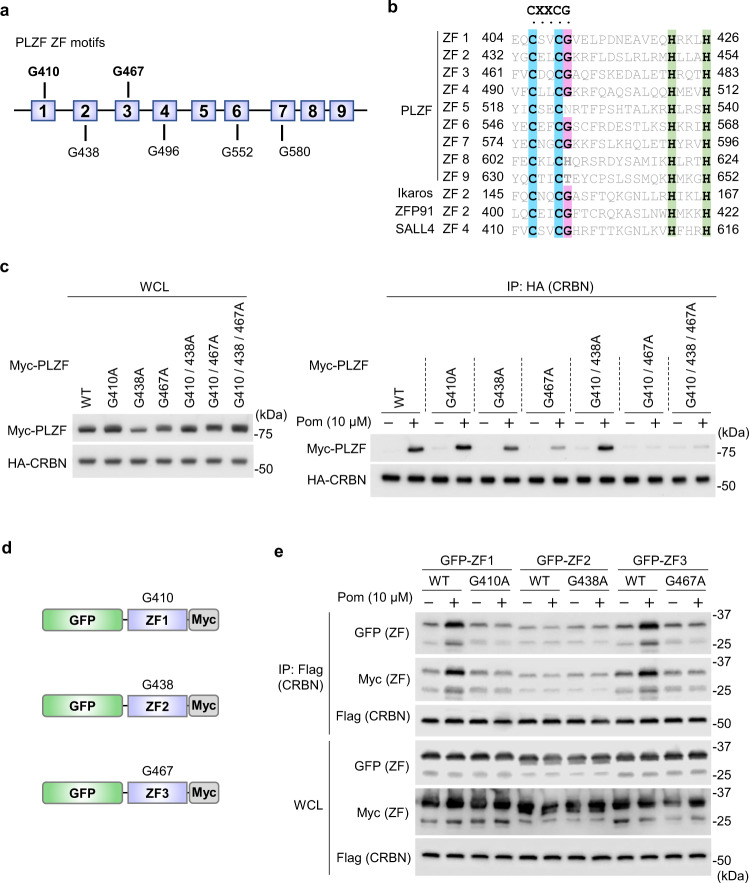
G410 and G467 of PLZF are critical for its binding to CRBN in the presence of pomalidomide. **a** Schematic representation of the ZF motifs of PLZF. **b** Multiple sequence alignment showing PLZF ZF motifs along with validated ZF degrons from Ikaros, ZFP91, and SALL4. Cys and His residues (blue and green, respectively) comprising ZF motifs and critical Gly residues (red) are highlighted. **c** FLAG-HA (FH)-CRBN and Myc-FLAG (MF)-PLZF carrying point mutations were coexpressed in 293T cells and immunoprecipitated with anti-HA antibody in the presence or absence of pomalidomide. **d** Schematic representation of EGFP-fused PLZF ZF proteins. **e** FH-CRBN and EGFP-fused PLZF ZF proteins were coexpressed in 293T cells and immunoprecipitated with anti-Flag antibody in the presence or absence of pomalidomide. WCL whole-cell lysate. All experiments were conducted more than twice with similar results.

PLZF is composed of the N-terminal BTB-POZ domain and nine C2H2-type ZF motifs (Fig. 3a)^29^. Recent reports have shown that many ZF-containing neosubstrates of CRBN have a CXXCG sequence motif(s) as the structural degron (X denotes any amino acid) and that the glycine residue is critical for their interactions with CRBN (Fig. 3b)^16–18,27^. Sequence analysis of PLZF found that six out of the nine ZFs possess a putative degron. We, therefore, constructed two PLZF mutants whose three glycine residues are replaced with alanine. While PLZF^G496/552/580A^ was coimmunoprecipitated with CRBN in a pomalidomide-dependent manner as efficiently as wild-type PLZF, PLZF^G410/438/467A^ lacked CRBN-binding activity (Supplementary Fig. 4a), and its expression was not downregulated by pomalidomide (Supplementary Fig. 4b). Next, single and double point mutants of PLZF were constructed based on the triple point mutant. Although all the single point mutants bound CRBN in a pomalidomide-dependent manner, PLZF^G410/467A^ did not bind CRBN appreciably (Fig. 3c and Supplementary Fig. 4c), suggesting that both G410 and G467 are critical for CRBN binding. Furthermore, a heterologous system using EGFP fused to a ZF motif of 23 amino acids was employed to validate the above finding (Fig. 3d). Binding experiments revealed that both ZF1 (404–426) and ZF3 (461–483) bind to CRBN in the presence of pomalidomide and the key glycine residue (Fig. 3e), leading us to conclude that PLZF has two structural degrons.

Some of the known neosubstrates of CRBN are resistant to degradation induced by CRBN-binding drugs in mice. Structural studies have shown that V388I substitution in mouse CRBN causes steric clash and prevents its interaction with neosubstrates^10,16,17^. We examined if pomalidomide induces degradation of PLZF in *CRBN*^−/−^ cells expressing mouse CRBN or human CRBN^V388I^. As a result, PLZF downregulation was observed in cells expressing human wild-type CRBN but not in cells expressing mouse CRBN or human CRBN^V388I^ (Supplementary Fig. 4d), suggesting that PLZF binds to CRBN with a stereospecificity similar to known neosubstrates containing a CXXCG motif.

### Pomalidomide-induced degradation of PLZF fusion proteins

**Fig. 4:**
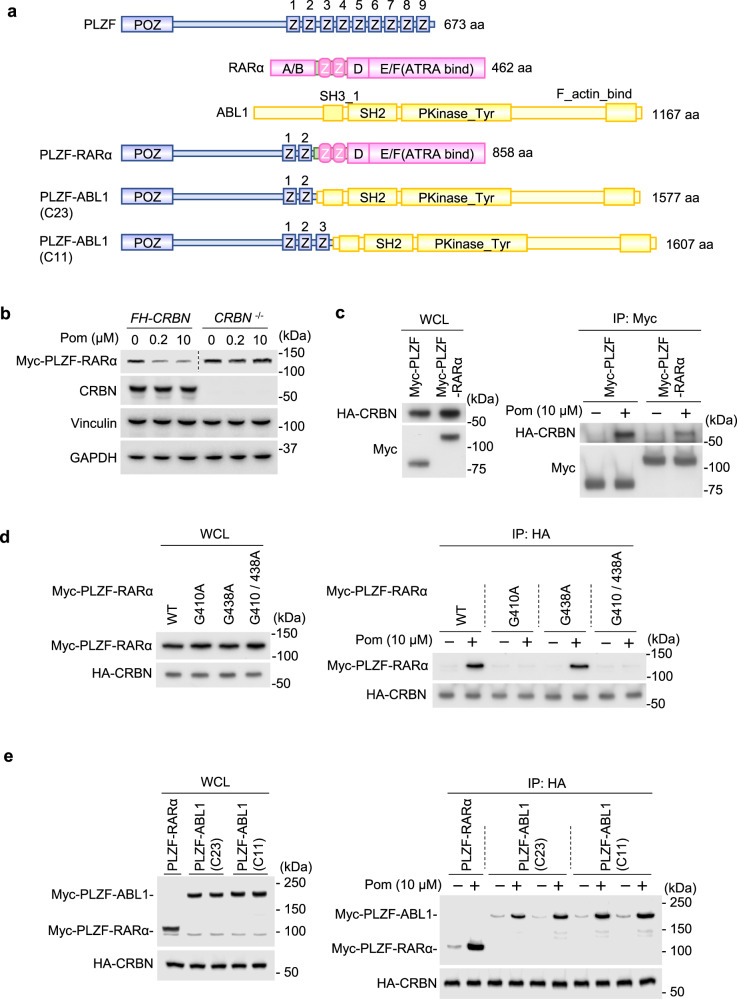
PLZF fusion proteins are neosubstrates of CRL4^CRBN^. **a** Schematic representation of PLZF, RARα, ABL1, and three PLZF fusion proteins. **b** Myc tagged-PLZF-RARα was overexpressed in 293T expressing FLAG-HA (FH)-CRBN and 293T lacking *CRBN*, treated with DMSO or pomalidomide, and subjected to immunoblot analysis. **c** FH-CRBN and Myc-FLAG (MF)-PLZF or Myc-PLZF-RARα were coexpressed in 293T cells and immunoprecipitated with anti-Myc antibody in the presence or absence of pomalidomide. **d** FH-CRBN and Myc-PLZF-RARα carrying point mutations were coexpressed in 293T cells and immunoprecipitated with anti-HA antibody in the presence or absence of pomalidomide. **e** FH-CRBN and Myc-PLZF-ABL1 were coexpressed and immunoprecipitated in the presence or absence of pomalidomide. WCL whole-cell lysate. All experiments were conducted more than twice with similar results.

PLZF is a transcription factor involved in multiple biological processes such as neurogenesis, spermatogenesis, invariant natural killer T-cell development, and leukemogenesis^25,30–32^. *PLZF* was originally identified as a gene that is fused to *RARα* following the t(11;17)(q23;q21) translocation in patients with APL (Fig. 4a)^33^. PLZF-RARα is associated with a rare subset of APL with a poor prognosis. If pomalidomide induces proteasomal degradation of PLZF-RARα at pharmacologically relevant concentrations, pomalidomide might be useful for the treatment of this type of APL. In addition, a recent study revealed that *PLZF* is fused with *ABL1* in two patients with T-ALL (C11 and C23)^26^. In all the cases identified so far, the N-terminal region of PLZF containing the first few ZF motifs is fused with partner gene products and plays a critical role in leukemogenesis (Fig. 4a). First, we investigated the stability of PLZF-RARα in the presence of pomalidomide. The PLZF-RARα protein level was decreased by 0.2 µM pomalidomide in 293T cells overexpressing PLZF-RARα, but not in the corresponding cells lacking *CRBN* (Fig. 4b). CRBN coimmunoprecipitated PLZF-RARα in the presence of pomalidomide (Fig. 4c and Supplementary Fig. 5a). The extent to which this interaction was stabilized by thalidomide or lenalidomide was weak, as observed for the CRBN–PLZF interaction (Supplementary Fig. 5a). Moreover, the mutational analysis showed that PLZF-RARα binding to CRBN is abolished by G410A mutation in ZF1, but not by G438A mutation in ZF2 (Fig. 4d), suggesting that PLZF-RARα binds to CRBN only through PLZF’s ZF1.

We also investigated the stability of PLZF-ABL1 and its interaction with CRBN in the presence or absence of pomalidomide. T-ALL cases C23 and C11 possess slightly different translocation breakpoints, resulting in the PLZF-ABL1 chimeric proteins retaining the first two or three zinc fingers of PLZF, respectively (Fig. 4a). CRBN coimmunoprecipitated the two PLZF-ABL1 isoforms at a comparable level in the presence of pomalidomide (Fig, 4e). Moreover, the PLZF-ABL1 protein levels, overexpressed in 293T cells, were reduced by pomalidomide treatment (Supplementary Fig. 5b), indicating that PLZF-ABL1 also serves as a pomalidomide-dependent neosubstrate of CRBN.

### Antiproliferative effect of pomalidomide on cells expressing PLZF-RARα

**Fig. 5:**
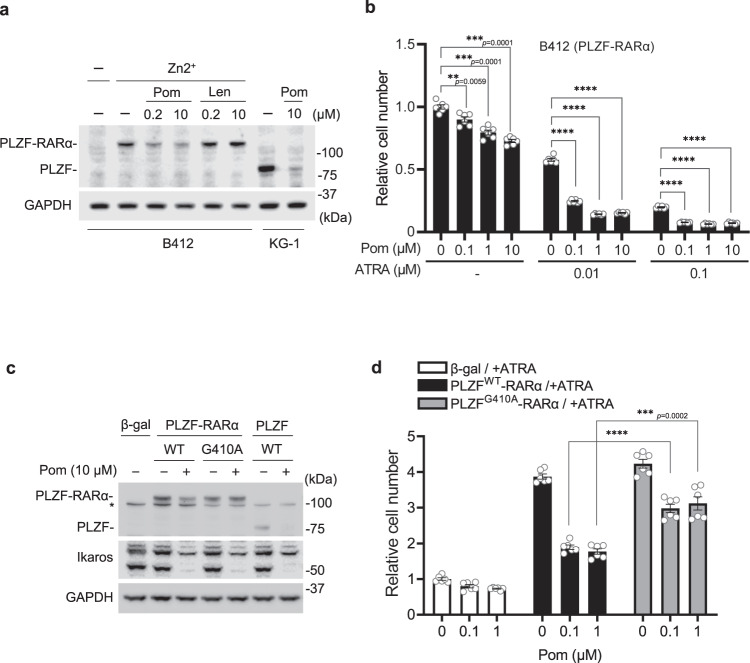
Antiproliferative effect of pomalidomide on cells expressing PLZF-RARα. **a** B412 and KG-1 cells were treated with lenalidomide or pomalidomide for 48 h. Where indicated, 100 μM ZnSO4 (Zn^2+^) was added 6 h prior to drug treatment. Cells were harvested and analyzed by immunoblotting. **b** The effect of pomalidomide on the proliferation of B412 cells. To B412 cells induced to express PLZF-RARα by ZnSO_4_, pomalidomide and ATRA were added 6 days prior to cell counting. Data are shown as mean ± SEM from six biologically independent samples. **p < 0.01; ***p < 0.001; ****p < 0.0001. Statistical significance was calculated with a two-way ANOVA with multiple comparisons test. **c** U937 cells expressing the indicated constructs were treated with pomalidomide or left untreated for 24 h, harvested, and subjected to immunoblotting. Asterisk denotes nonspecific signal. **d** U937 cells expressing the indicated constructs were treated with ATRA and the indicated concentrations of pomalidomide for 7 days prior to cell counting. Data are shown as mean ± SEM from six biologically independent experiments. ***p < 0.001; ****p < 0.0001. Statistical significance was calculated with two-way ANOVA with multiple comparisons test. Experiments were conducted more than twice with similar results.

Next, we employed B412, a U937-derived stable clone with zinc-inducible expression of PLZF-RARα, which has been used as a cell line model of PLZF-RARα-positive APL in many studies^34–36^. Zinc-induced PLZF-RARα expression in B412 cells was downregulated by pomalidomide, but not by lenalidomide (Fig. 5a and Supplementary Fig. 5c). To investigate whether pomalidomide exerts its antiproliferative effect through the degradation of PLZF-RARα, B412 cells were treated with pomalidomide, all-*trans* retinoic acid (ATRA), or both for 6 days and subjected to tetrazolium-based assays for cell viability. ATRA alone inhibited B412 cell growth in a concentration-dependent manner (Fig. 5b). Pomalidomide alone also showed a modest inhibitory effect. Remarkably, in the presence of a suboptimal concentration of ATRA, pomalidomide showed a more prominent inhibitory effect at pharmacologically relevant concentrations (Fig. 5b). This finding led us to investigate whether ATRA influences the interaction between CRBN and PLZF-RARα. In fact, however, ATRA had little effect on their interaction (Supplementary Fig. 5d), suggesting that ATRA and pomalidomide exert their antiproliferative effects by distinct mechanisms.

To determine whether the antiproliferative effect of pomalidomide on B412 cells is mediated by CRBN binding to ZF1 of PLZF-RARα, U937 cells constitutively expressing PLZF^WT^-RARα, PLZF^G410A^-RARα, PLZF^WT^, or control β-galactosidase were established by using a lentiviral vector. While PLZF^WT^-RARα, PLZF^G410A^-RARα, and PLZF^WT^ were expressed at a similar level in the absence of pomalidomide, only PLZF^WT^-RARα and PLZF^WT^ were substantially downregulated by pomalidomide (Fig. 5c). Forced expression of PLZF^WT^-RARα or PLZF^G410A^-RARα resulted in an approximately four-fold increase in cell number after 7 days of incubation (Fig. 5d), demonstrating the growth-promoting activity of the fusion protein. The growth-promoting activity of PLZF^G410A^-RARα was not efficiently reversed by pomalidomide, whereas that of PLZF^WT^-RARα was reversed by pomalidomide (Fig. 5d), indicating that CRBN binding to PLZF-RARα and its destruction is responsible for the antiproliferative effect of pomalidomide.

## Discussion

This study illustrates that PLZF and its fusion proteins are pomalidomide-dependent neosubstrates of CRL4^CRBN^. PLZF is susceptible to proteasomal degradation in the presence of pomalidomide, but not in the presence of thalidomide or lenalidomide. PLZF possesses two degrons in ZF1 (404–426) and ZF3 (461–483). PLZF-RARα and PLZF-ABL1, fusion proteins associated with APL and T-ALL, respectively, are eliminated by pomalidomide at pharmacologically relevant concentrations. In addition, pomalidomide exerts an antiproliferative effect on U937 cells expressing PLZF-RARα.

**Fig. 6:**
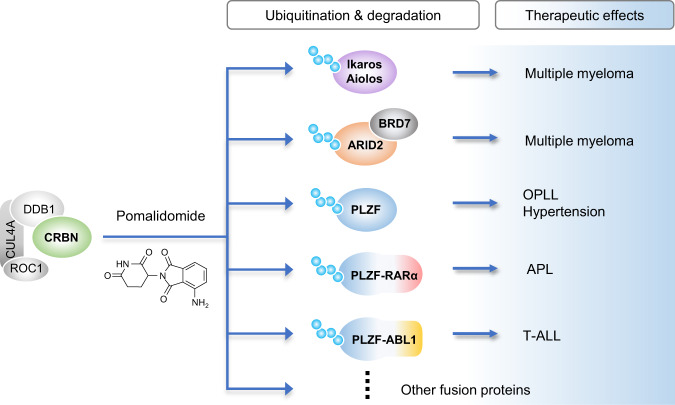
Schematic representation of pomalidomide effects. In multiple myeloma, pomalidomide induces the degradation of Ikaros, Aiolos, and ARID2, resulting in anti-myeloma effects. Pomalidomide also induces degradation of PLZF and may exert several physiological and pathological effects via its breakdown. In APL and T-ALL expressing PLZF fusion proteins, pomalidomide may exert an anti-cancer effect by inducing the degradation of the leukemogenic fusion proteins.

APL is characterized by excessive proliferation of immature leukemic cells harboring chromosomal translocations between *RARα* and one of several partner genes^25^. The vast majority of APL patients bear PML-RARα and respond to ATRA therapy. On the other hand, APL patients bearing PLZF-RARα are resistant to ATRA-induced differentiation and have poor prognosis^25,37^. Given the growth-promoting activity of PLZF-RARα^35^, targeted degradation of PLZF-RARα is a promising therapeutic approach against refractory APL carrying PLZF-RARα. Our data have shown that pomalidomide, an immunomodulatory drug approved for the treatment of multiple myeloma, is an attractive candidate for a PLZF-RARα degrader (Fig. 6).

PROTACs or molecular glue degraders targeting PLZF may also be useful for the treatment of other diseases. One such example is ossification of the posterior longitudinal ligament (OPLL), a disease-causing cervical myopathy that is characterized by ectopic bone formation in the paravertebral ligament^38,39^ A previous study reported that PLZF is up-regulated in OPLL cells and that knockdown of PLZF leads to downregulation of osteoblast-specific genes and inhibition of osteoblastic differentiation^39^. Concordantly, a following study showed that PLZF is recruited to the enhancers of osteogenic genes and activates nearby genes through epigenetic regulation^40^. Thus, pomalidomide may exert therapeutic effects on OPLL via degradation of PLZF (Fig. 6). Other studies have shown that PLZF is associated with metabolic and cardiac traits of spontaneously hypertensive rats (SHRs)^41–43^. SHR is one of the most widely used model of essential hypertension, which likely develops left ventricular hypertrophy, myocardial fibrosis, and metabolic disturbances. Previous studies have shown that PLZF mediates cardiac hypertrophic signaling and that its downregulation ameliorates the metabolic and cardiac traits of the animals^41,43^. Thus, pomalidomide or other PROTACs targeting PLZF using pomalidomide or its derivatives may also be useful for the treatment of metabolic syndrome and cardiovascular diseases (Fig. 6).

During the preparation of this paper, one report independently revealed that PLZF is a neosubstrate involved in thalidomide teratogenicity^44^. Using a human transcription factor protein array, the authors identified PLZF as a neosubstrate whose degradation is induced by thalidomide and its derivatives. Meanwhile, here we investigated potential repositioning of thalidomide analogs to leukemias associated with *PLZF* translocation. Thus, these two studies are completely different beyond the identification of PLZF as a neosubstrate for CRL4^CRBN^. According to Yamanaka et al.^44^, pomalidomide is most potent in inducing the degradation of PLZF among approved thalidomide-based compounds, followed by lenalidomide and thalidomide. In our hands, while pomalidomide is most potent, the efficacy of thalidomide and lenalidomide is very low, if not nonexistent (Fig. 1c and Supplementary Fig. 2e). This is a minor conflict between the two studies. Drug concentration and incubation time were almost the same, but different cell lines were used by the two studies. Thus, a possible explanation for the apparent discrepancy is that the cell lines used in these studies had different ubiquitin-proteasome system activity.

There are numerous fusion genes driving cancers by translocations. Pharmaceutical drugs that target fusion gene products have been developed. One of the most well-known drugs in this category is imatinib, which targets BCR-ABL in chronic myeloid leukemia^45^. While kinases and receptors are comparatively easy to be functionally inactivated by small-molecule compounds and are considered druggable, transcription factors and signal transducers are difficult to be targeted by conventional approaches and are considered undruggable^46^. Recently, however, proteolysis targeting chimeras (PROTACs) and molecular glue degraders^47^ have been investigated as a strategy for drug development. Ikaros and Aiolos, CRBN neosubstrates and transcription factors essential for myeloma survival, are conventionally undruggable but are now regarded as promising targets for drug development^48^. Similarly, oncogenic fusion proteins without enzymatic activity can also be targeted by PROTACs or molecular glue degraders. Meanwhile, recent studies using massive parallel sequencing and clinical samples have led to the identification of numerous fusion genes^26,49^. For example, from T-ALL patients, fusion genes, such as PLZF-ABL1 and IKZF1-Notch1, have been found^26,50^. Since the PLZF moiety of PLZF-ABL1 and the IKZF1 moiety of IKZF1-Notch1 carry a structural degron for CRL4^CRBN^, we speculate that pomalidomide and lenalidomide may also be effective for the treatment of T-ALL carrying these fusion proteins. Thus, PROTAC-based degraders targeting proteins that are frequently mutated and fused to other proteins by chromosomal translocations may serve as small-molecule drugs for treating multiple diseases caused by common genetic aberrations. At least the approved drug pomalidomide is worth investigating for use in PLZF-associated APL and T-ALL. This study highlights a potential therapeutic use of pomalidomide for the treatment of cancers carrying neosubstrate-fused oncogenic drivers (Fig. 6).

## Methods

### Cell culture

Human embryonic kidney cell line 293T (ATCC) was maintained in Dulbecco’s modified Eagle’s medium (Nacalai Tesque) supplemented with 10% fetal bovine serum (Biowest) and Antibiotic-Antimycotic Mixed Stock solution (Nacalai Tesque). Neural stem cell lines AF22 (lt-NES) and Sai2 were cultured on Matrigel (Corning, 354277) and maintained in RHB-A medium (CEL, Y40001) supplemented with 8 µL/mL Stem Beads FGF2 (Stem Culture Inc, SB500) and 10 ng/mL EGF (PEPROTECH, AF-100). KG1 and U937 cells were maintained in RPMI1640 (ThermoFisher Scientific, A1049101) containing 10% fetal bovine serum. All the cell lines were cultured at 37 °C with 5% CO_2_ and were confirmed to be mycoplasma-negative using the MycoAlert Mycoplasma Detection Kit (Lonza).

### Reagents

Thalidomide (Toris Biosciences, 0652), pomalidomide (TCI chemicals, 19171-19-8), MLN4924 (Adooq BioScience, 905579-51-3), and all-*trans* retinoic acid (SIGMA, R2625) were purchased from the indicated vendors. Lenalidomide was a kind gift from Celgene.

### Plasmids

pCMV6-PLZF-MF and pCMV6-SALL4-MF were purchased from ORIGENE. PLZF-RARα and PLZF-ABL1 cDNAs were synthesized by Eurofins Genomics and GenScript, respectively, and inserted to pCS2(+) and pLenti6. PLZF and PLZF-RARα mutants were generated by using PrimeSTAR Mutagenesis Basal Kit (TaKaRa). pcDNA3.1-FH-tagged human CRBN was described previously^5^.

### Mass spectrometry

293T cells expressing FH-CRBN were established by lentiviral transduction. Cells (1 × 10^8^) were collected in 1% Triton lysis buffer (50 mM Tris-HCl pH 8.0, 150 mM NaCl, and 1% Triton X-100) supplemented with protease/phosphatase inhibitor cocktail. The cell lysate was mixed with M2 FLAG agarose (Sigma, A2220) and incubated for 3 h. After washing the beads six times with 1% Triton lysis buffer, bound proteins (CRL4^FH-CRBN^) were eluted with 0.1 mg/mL FLAG peptide. Lt-NES cells (5 × 10^8^) were extracted using 1% Triton lysis buffer containing protease/phosphatase inhibitor cocktail, and the extracts were combined with the CRL4^FH-CRBN^ complex and 0.1% DMSO or 10 μM pomalidomide. After 12 h of incubation, the mixture was incubated with Anti-HA-Affinity Matrix beads (Roche, 11815016001) for 3 h. After washing the beads five times with 1% Triton lysis buffer, bound proteins were eluted with sodium dodecyl sulfate (SDS) and subjected to SDS-PAGE following Coomassie Brilliant Blue (CBB) staining. Gels were sliced and sent to Medical ProteoScope Co., Ltd. Tryptic peptides were extracted from the slices and subjected to the Ultimate 3000 RSLCnano liquid chromatography (ThermoFisher Scientific) and the Q Exactive Orbitrap mass spectrometer (ThermoFisher Scientific). Data were analyzed by Proteome Discoverer (ver. 2.2) (ThermoFisher Scientific) and Mascot (Matrix Science).

### Coimmunoprecipitation assay

For coimmunoprecipitation analysis of exogenous proteins, N-terminally FH-tagged CRBN and C-terminally MF-tagged PLZF were transfected into 293T cells using Lipofectamine 2000 (ThermoFisher Scientific). Cells were collected and extracted by 1% Triton lysis buffer supplemented with protease/phosphatase inhibitor cocktail 48 h after transfection. Extracts were incubated for 12 h in the presence or absence of pomalidomide or its analogs. The mixture was then incubated with anti-HA affinity matrix beads for 3 h. After washing the beads five times with 1% Triton lysis buffer, bound proteins were eluted with SDS and analyzed by immunoblotting.

For coimmunoprecipitation of endogenous proteins, extracts of lt-NES cells were incubated with mouse monoclonal anti-CRBN44 antibody, generated against amino acids 1–18 of human CRBN^51^, for 24 h in the presence or absence of pomalidomide. The mixture was then incubated with Dynabeads Protein G (Veritas) for 3 h. After washing the beads five times with 1% Triton lysis buffer, bound proteins were eluted with SDS and analyzed by immunoblotting.

### CRISPR/Cas9-mediated genome editing

For the generation of lt-NES *CRBN*^−^^/−^ and HEK293T *CRBN*^−^^/−^ cells, parental cells were transfected with 2 µg of spCas9-sgRNA targeting *CRBN* using Nucleofector kit V (Lonza) and program B-23. After transfection, clonal isolation was conducted. The protein level of CRBN in each cell line was assessed by immunoblotting. CRBN guide RNA sequence used was 5′-GCTGATATGGAAGAATTTCATGG-3′.

### Immunoblotting

Samples were subjected to 7% or 10% SDS-PAGE (DRC XV PANTERA GEL, NXV-225P) and transferred to PVDF membranes using the Trans-Blot Turbo Transfer System (Bio-Rad). Membranes were blocked with Bullet Blocking One (Nacalai Tesque) and incubated with primary antibodies overnight, followed by three washes with 0.1% TBST and incubation with secondary antibodies for 45 min. After three final washes, the membranes were incubated with Chemi-Lumi One L (Nacalai Tesque, 07880-54) or Chemi-Lumi One Ultra (Nacalai Tesque, 11644-40) for 2 min and subjected to imaging with FUSION FX (Vilber-Lourmat). Rabbit anti-human CRBN65 mAb (Celgene, San Diego, CA, 1:10000 dilution, Lot# CGN-6-4-5), mouse anti-PLZF mAb (39987, Active Motif, 1:1000 dilution, Lot#05313002), mouse anti-PLZF mAb (sc-28319, Santa Cruz, 1:1000 dilution, Lot#C0618), mouse anti-SALL4 mAb (ab57577, Abcam, 1:1000 dilution, Lot#GR3198290-2), mouse anti-Vinculin mAb (ab18058, Abcam, 1:1000 dilution, Lot#GR207163-1), mouse anti-HA11 mAb (901503, BioLegend, 1:1000 dilution, Lot#b207273), mouse anti-Flag M2 mAb (F1804, SIGMA, 1:1000 dilution, Lot#SLBS3530V), rabbit anti-Myc tag (ab9106, Abcam, 1:1000 dilution), rabbit anti-GSPT1 (ab49878, Abcam, 1:1000 dilution, Lot#GR274469-4), rabbit anti-CK1α mAb (ab108296, Abcam, 1:1000 dilution, Lot#GR53415-9), and rabbit anti-Ikaros mAb (ab26083, Abcam, 1:1000 dilution, Lot#GR250435-1), rabbit anti-GFP (ab290, Abcam, 1:1000 dilution, Lot#GR3184825-1) were used as primary antibodies. Anti-mouse IgG, HRP-linked Antibody (#7076, Cell Signaling, 1:10,000 dilution, Lot#25) and anti-rabbit IgG, HRP-linked Antibody (#7074, Cell Signaling, 1:10000 dilution, Lot#33) were used as secondary antibodies. Mouse anti-GAPDH-mAb-HRP-DirecT (M171-7, MBL, 1:5000 dilution, Lot#006), mouse anti-Myc-tag mAb-HRP-DirecT (M192-7, MBL, 1:2000 dilution, Lot#006), mouse anti-Ub (P4D1)-mAb-HRP (sc-8017, Santa Cruz, 1:1000 dilution, Lot#A0919), anti-Ub mAb (FK2, ENZO, 1:1000 dilution, Lot#07281715), and rabbit anti-GST-tag pAb-HRP-DirecT (PM013-7, MBL, 1:1000 dilution, Lot#005) were also used.

### Immunofluorescence microscopy

Lt-NES cells were grown on Matrigel-coated 8-well chamber slides (5732-008, IWAKI) for 24 h and further incubated in the presence of DMSO or 10 μM pomalidomide for 24 h. Then, the media was discarded, and the wells were washed once with PBS. Cells were fixed with 4% paraformaldehyde in PBS for 20 min, washed three times for 5 min with PBS containing 0.1% Triton X-100, and then blocked for 1 h at room temperature with PBS containing 10% FBS. Anti-PLZF (39987, Active Motif, Lot#05313002) and anti-SOX2 (ab92494, Abcam, Lot#GR30587-10) were diluted at 1:100 with PBS containing 10% FBS (antibody dilution buffer) and incubated overnight. After three-time washes with PBS, Alexa Fluor 488 donkey anti-mouse (Life Technologies) and Alexa Fluor 555 anti-rabbit (Life Technologies) antibodies diluted at 1:500 with antibody dilution buffer were added and incubated for 1 h at room temperature. After three-time washes with PBS, coverslips were mounted onto the slides with Aqueous Mounting Medium (TA-030-FM, ThermoFisher Scientific) containing Hoechst 33258 (ab228550, Abcam). Slides were analyzed by fluorescence microscopy at ×20 using an EVOS Imaging System. Fluorescence intensities were quantified by using ImageJ.

### Quantitative RT-PCR analysis

From lt-NES cells treated with 10 µM pomalidomide or DMSO, RNA was isolated using Sepasol reagent (Nacalai Tesque) and reverse-transcribed using iScript RT Supermix (Bio-Rad) following the manufacturer’s instructions. The following primer sets (Eurofins) were used with SsoAdvanced Universal SYBR Green Supermix (Bio-Rad) and a CFX96 Real-Time PCR System (Bio-Rad) to quantify *GAPDH* and *PLZF* mRNA levels. GAPDH, 5′-GGATTTGGTCGTATTGGG-3′ and 5′-GGAAGATGGTGATGGGATT-3′; PLZF, 5′-CGGGACTTTGTGCGATGTG-3′ and 5′-GCGGTGGAAGAGGATCTCAA-3′. Relative mRNA levels were calculated by using the ΔΔCT method.

### GST-pulldown assay

For binding experiments with purified proteins, Flag-tagged PLZF (Active Motif) and GST-tagged CRBN (Novus Biologicals) were incubated with 10 μM thalidomide, pomalidomide, or lenalidomide in binding buffer (25 mM Tris-HCl pH 7.5, 150 mM NaCl, 1 mM EDTA, 10% glycerol, and 1% Triton X-100) containing 0.1 mg/mL BSA. After incubation at 4 °C for 20 h, mixtures were further incubated with glutathione Sepharose beads (GE Healthcare) for 3 h. After washing the beads three times, bound proteins were eluted by SDS and analyzed by immunoblotting.

### PLZF protein half-life analysis

Sai2 cells were pretreated with DMSO or 1 µM pomalidomide for 30 min, followed by the addition of 100 μg/mL cycloheximide (Nacalai Tesque) to the culture medium. At various time points, cells were collected and subjected to immunoblot analysis.

### Cellular ubiquitination assays

To assess endogenous ubiquitination of PLZF and SALL4, 2 × 10^7^ lt-NES cells were pretreated with DMSO or 1 μM MLN4924, treated with DMSO or 10 μM pomalidomide for 7 h, and then lysed with Pierce IP Lysis Buffer (ThermoFisher Scientific) containing 10 mM *N*-ethylmaleimide (ThermoFisher Scientific), 10 μM PR-619 (Nacalai Tesque), 10 μM MG132 (Peptide Institute), and protease/phosphatase inhibitor cocktail. Ubiquitinated proteins were pulled down by incubating lysates with Ubiquilin 1 tandem UBA (TUBE2) agarose (Boston Biochem) for 2 h at 4 °C and washing the beads 3 times with IP lysis buffer. Bound proteins were eluted by incubating the beads with Sample Buffer Solution with Reducing Reagent (6×) for SDS-PAGE (Nacalai Tesque) at 95 °C for 5 min and separated by SDS-PAGE, followed by immunoblotting using antibodies against PLZF, SALL4, and Ubiquitin.

### In vitro ubiquitination assay

Flag-tagged recombinant PLZF (Active Motif) and recombinant CRL4^CRBN^ complex were pre-incubated with DMSO or 100 µM pomalidomide in 1× E3 Ligase Reaction Buffer (R&D Systems) for 30 min at 30 °C. The reactions were then incubated with 100 nM UBE1 (E1; R&D Systems), 500 nM UBE2D3 (E2; R&D Systems), 500 nM UBE2G1 (E2; R&D Systems), 25 µM ubiquitin (R&D Systems), and 2 µM ubiquitin-aldehyde (R&D Systems) in the presence or absence of 1 mM Mg-ATP (R&D Systems) for 90 min at 30 °C. Flag-PLZF was then isolated using anti-Flag affinity gel (Sigma) and subjected to immunoblotting to visualize ubiquitinated PLZF using an anti-ubiquitin antibody (Enzo Life Science).

### Cell proliferation analysis

Cells were plated into a 96-well plate and incubated with Cell Count Reagent SF (Nacalai Tesque) for 1–3 h. Then, relative cell numbers were estimated by measuring the absorbance at 450 and 600 nm.

### Statistics and reproducibility

Quantitative data were expressed in bar graphs or scatter plots as mean ± standard error of the mean (SEM). The number of tests carried out is stated in figure legends. The data were calculated and analyzed by Microsoft Excel (Microsoft), Prism 8 (GraphPad Software).

### Reporting summary

Further information on research design is available in the Nature Research Reporting Summary linked to this article.

## Supplementary information

Supplementary Information

Supplementary Data 1

Supplementary Data 2

Reporting Summary

## Data Availability

All data generated or analyzed during this study are included in this published article (and its Supplementary Information files) or are available from the corresponding author on reasonable request. The source data underlying the graphs and charts in the figure are shown in Supplementary Data 2. Proteomics raw data are deposited in the Japan Proteome Standard Repository/Database (jPOST), a member of the ProteomeXchange consortium. The accession numbers are PXD023928 for ProteomeXchange and JPST001075 for jPOST.

## References

[1] Bartlett JB, Dredge K, Dalgleish AG (2004). The evolution of thalidomide and its IMiD derivatives as anticancer agents. Nat. Rev. Cancer.

[2] Adams J (1998). Potent and selective inhibitors of the proteasome: dipeptidyl boronic acids. Bioorg. Med. Chem. Lett..

[3] Ito T, Handa H (2020). Molecular mechanisms of thalidomide and its derivatives. Proc. Jpn Acad. Ser. B Phys. Biol. Sci..

[4] Singhal S (1999). Antitumor activity of thalidomide in refractory multiple myeloma. N. Engl. J. Med..

[5] Ito T (2010). Identification of a primary target of thalidomide teratogenicity. Science.

[6] Ito T, Handa H (2015). Myeloid disease: another action of a thalidomide derivative. Nature.

[7] Lu G (2014). The myeloma drug lenalidomide promotes the cereblon-dependent destruction of Ikaros proteins. Science.

[8] Kronke J (2014). Lenalidomide causes selective degradation of IKZF1 and IKZF3 in multiple myeloma cells. Science.

[9] Gandhi AK (2014). Immunomodulatory agents lenalidomide and pomalidomide co-stimulate T cells by inducing degradation of T cell repressors Ikaros and Aiolos via modulation of the E3 ubiquitin ligase complex CRL4(CRBN.). Br. J. Haematol..

[10] Kronke J (2015). Lenalidomide induces ubiquitination and degradation of CK1alpha in del(5q) MDS. Nature.

[11] Yamamoto J (2020). ARID2 is a pomalidomide-dependent CRL4(CRBN) substrate in multiple myeloma cells. Nat. Chem. Biol..

[12] Asatsuma-Okumura T (2019). p63 is a cereblon substrate involved in thalidomide teratogenicity. Nat. Chem. Biol..

[13] Donovan KA (2018). Thalidomide promotes degradation of SALL4, a transcription factor implicated in Duane Radial Ray syndrome. Elife.

[14] Matyskiela ME (2018). SALL4 mediates teratogenicity as a thalidomide-dependent cereblon substrate. Nat. Chem. Biol..

[15] Belair DG (2020). Thalidomide inhibits human iPSC mesendoderm differentiation by modulating CRBN-dependent degradation of SALL4. Sci. Rep..

[16] Matyskiela ME (2016). A novel cereblon modulator recruits GSPT1 to the CRL4(CRBN) ubiquitin ligase. Nature.

[17] Petzold G, Fischer ES, Thoma NH (2016). Structural basis of lenalidomide-induced CK1alpha degradation by the CRL4(CRBN) ubiquitin ligase. Nature.

[18] Sievers QL (2018). Defining the human C2H2 zinc finger degrome targeted by thalidomide analogs through CRBN. Science.

[19] Matyskiela ME (2020). Crystal structure of the SALL4-pomalidomide-cereblon-DDB1 complex. Nat. Struct. Mol. Biol..

[20] Tan X (2007). Mechanism of auxin perception by the TIR1 ubiquitin ligase. Nature.

[21] List A (2006). Lenalidomide in the myelodysplastic syndrome with chromosome 5q deletion. N. Engl. J. Med..

[22] Habermann TM (2009). Lenalidomide oral monotherapy produces a high response rate in patients with relapsed or refractory mantle cell lymphoma. Br. J. Haematol..

[23] Ramsay AG (2009). Follicular lymphoma cells induce T-cell immunologic synapse dysfunction that can be repaired with lenalidomide: implications for the tumor microenvironment and immunotherapy. Blood.

[24] Rychak E (2016). Pomalidomide in combination with dexamethasone results in synergistic anti-tumour responses in pre-clinical models of lenalidomide-resistant multiple myeloma. Br. J. Haematol..

[25] Melnick A, Licht JD (1999). Deconstructing a disease: RARalpha, its fusion partners, and their roles in the pathogenesis of acute promyelocytic leukemia. Blood.

[26] Chen B (2018). Identification of fusion genes and characterization of transcriptome features in T-cell acute lymphoblastic leukemia. Proc. Natl Acad. Sci. USA.

[27] An J (2017). pSILAC mass spectrometry reveals ZFP91 as IMiD-dependent substrate of the CRL4(CRBN) ubiquitin ligase. Nat. Commun..

[28] Davies, F. & Baz, R. Lenalidomide mode of action: linking bench and clinical findings. *Blood Rev*. **Suppl 1**, S13-S19 (2010). 10.1016/S0268-960X(10)70004-7 21126632

[29] Ahmad KF, Engel CK, Prive GG (1998). Crystal structure of the BTB domain from PLZF. Proc. Natl Acad. Sci. USA.

[30] Cook M (1995). Expression of the zinc-finger gene PLZF at rhombomere boundaries in the vertebrate hindbrain. Proc. Natl Acad. Sci. USA.

[31] Costoya JA (2004). Essential role of Plzf in maintenance of spermatogonial stem cells. Nat. Genet..

[32] Kovalovsky D (2008). The BTB-zinc finger transcriptional regulator PLZF controls the development of invariant natural killer T cell effector functions. Nat. Immunol..

[33] Chen Z (1993). Fusion between a novel Kruppel-like zinc finger gene and the retinoic acid receptor-alpha locus due to a variant t(11;17) translocation associated with acute promyelocytic leukaemia. EMBO J..

[34] Muller C (2000). The aberrant fusion proteins PML-RAR alpha and PLZF-RAR alpha contribute to the overexpression of cyclin A1 in acute promyelocytic leukemia. Blood.

[35] Rice KL (2009). Comprehensive genomic screens identify a role for PLZF-RARalpha as a positive regulator of cell proliferation via direct regulation of c-MYC. Blood.

[36] Ruthardt M (1998). The acute promyelocytic leukaemia specific PML and PLZF proteins localize to adjacent and functionally distinct nuclear bodies. Oncogene.

[37] Wang X, Wang J, Zhang L (2019). Characterization of atypical acute promyelocytic leukaemia: Three cases report and literature review. Medicine.

[38] Smith ZA, Buchanan CC, Raphael D, Khoo LT (2011). Ossification of the posterior longitudinal ligament: pathogenesis, management, and current surgical approaches. A review. Neurosurg. Focus.

[39] Ikeda R (2005). The promyelotic leukemia zinc finger promotes osteoblastic differentiation of human mesenchymal stem cells as an upstream regulator of CBFA1. J. Biol. Chem..

[40] Agrawal Singh S (2019). PLZF targets developmental enhancers for activation during osteogenic differentiation of human mesenchymal stem cells. Elife.

[41] Liska F (2014). Plzf as a candidate gene predisposing the spontaneously hypertensive rat to hypertension, left ventricular hypertrophy, and interstitial fibrosis. Am. J. Hypertens..

[42] Liska F (2017). Downregulation of Plzf gene ameliorates metabolic and cardiac traits in the spontaneously hypertensive rat. Hypertension.

[43] Wang N (2012). Promyelocytic leukemia zinc finger protein activates GATA4 transcription and mediates cardiac hypertrophic signaling from angiotensin II receptor 2. PLoS ONE.

[44] Yamanaka S (2021). Thalidomide and its metabolite 5-hydroxythalidomide induce teratogenicity via the cereblon neosubstrate PLZF. EMBO J..

[45] Druker BJ (1996). Effects of a selective inhibitor of the Abl tyrosine kinase on the growth of Bcr-Abl positive cells. Nat. Med..

[46] Dang CV, Reddy EP, Shokat KM, Soucek L (2017). Drugging the ‘undruggable’ cancer targets. Nat. Rev. Cancer.

[47] Lai AC, Crews CM (2017). Induced protein degradation: an emerging drug discovery paradigm. Nat. Rev. Drug Discov..

[48] Fionda C (2015). The IMiDs targets IKZF-1/3 and IRF4 as novel negative regulators of NK cell-activating ligands expression in multiple myeloma. Oncotarget.

[49] Maher CA (2009). Transcriptome sequencing to detect gene fusions in cancer. Nature.

[50] Girardi T, Vicente C, Cools J, De Keersmaecker K (2017). The genetics and molecular biology of T-ALL. Blood.

[51] Lopez-Girona A (2012). Cereblon is a direct protein target for immunomodulatory and antiproliferative activities of lenalidomide and pomalidomide. Leukemia.

