# Supplementary material for: PLZF and its fusion proteins are pomalidomide-dependent CRBN neosubstrates
Source: Commun Biol. 2021 Nov 11;4:1277. doi: 10.1038/s42003-021-02801-y (PMC8586336; doi:10.1038/s42003-021-02801-y)
Supplement: Supplementary file 1 — Supplementary Information [file 42003_2021_2801_MOESM1_ESM.pdf]

Supplementary Materials for

PLZF and its fusion proteins are pomalidomide-dependent CRBN neosubstrates.

Nobuyuki Shimizu[1], Tomoko Asatsuma-Okumura[1], Junichi Yamamoto[2], Yuki Yamaguchi[2], Hiroshi Handa[1†] and Takumi Ito[1†].

[1]Department of Chemical Biology, Tokyo Medical University, 6-1-1, Shinjuku, Shinjuku-ku, Tokyo 160-8402, Japan

[2]School of Life Science and Technology, Tokyo Institute of Technology, Yokohama 226-8501, Japan

†Corresponding authors.

E-mail: hhanda@tokyo-med.ac.jp

E-mail: takumii@tokyo-med.ac.jp

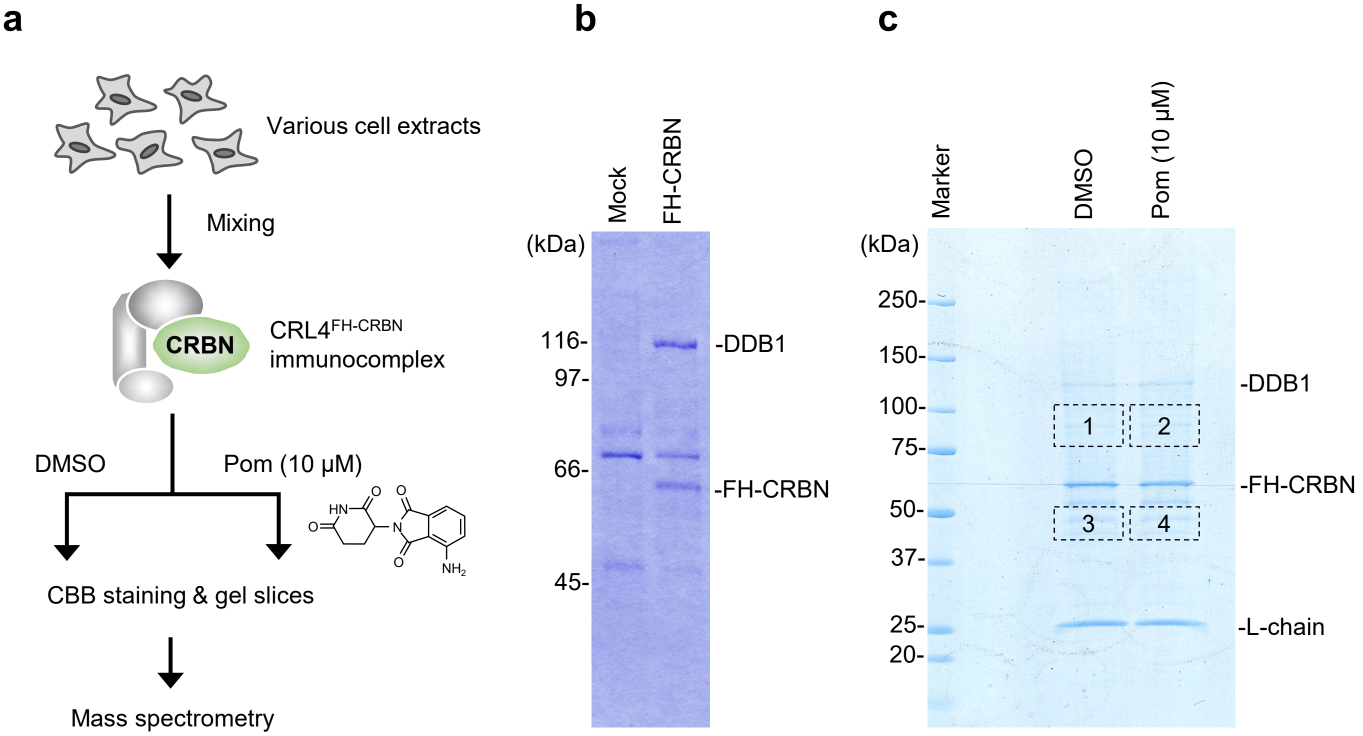

Supplementary Figure 1. Identification of pomalidomide-dependent CRBN-binding proteins.

a. Scheme of isolation of pomalidomide-dependent CRBN-binding proteins. **b.** The $CRL4^{FH\text{-}CRBN}$ complex was purified from 293T cells stably expressing FH-CRBN and stained with CBB following SDS-PAGE. **c.** The purified complex was incubated with various cell extracts for 24 h in the presence or absence of pomalidomide. Bound proteins were then affinity-purified using anti-HA beads and subjected to SDS-PAGE and CBB staining. Gel slices were prepared and subjected to MS.

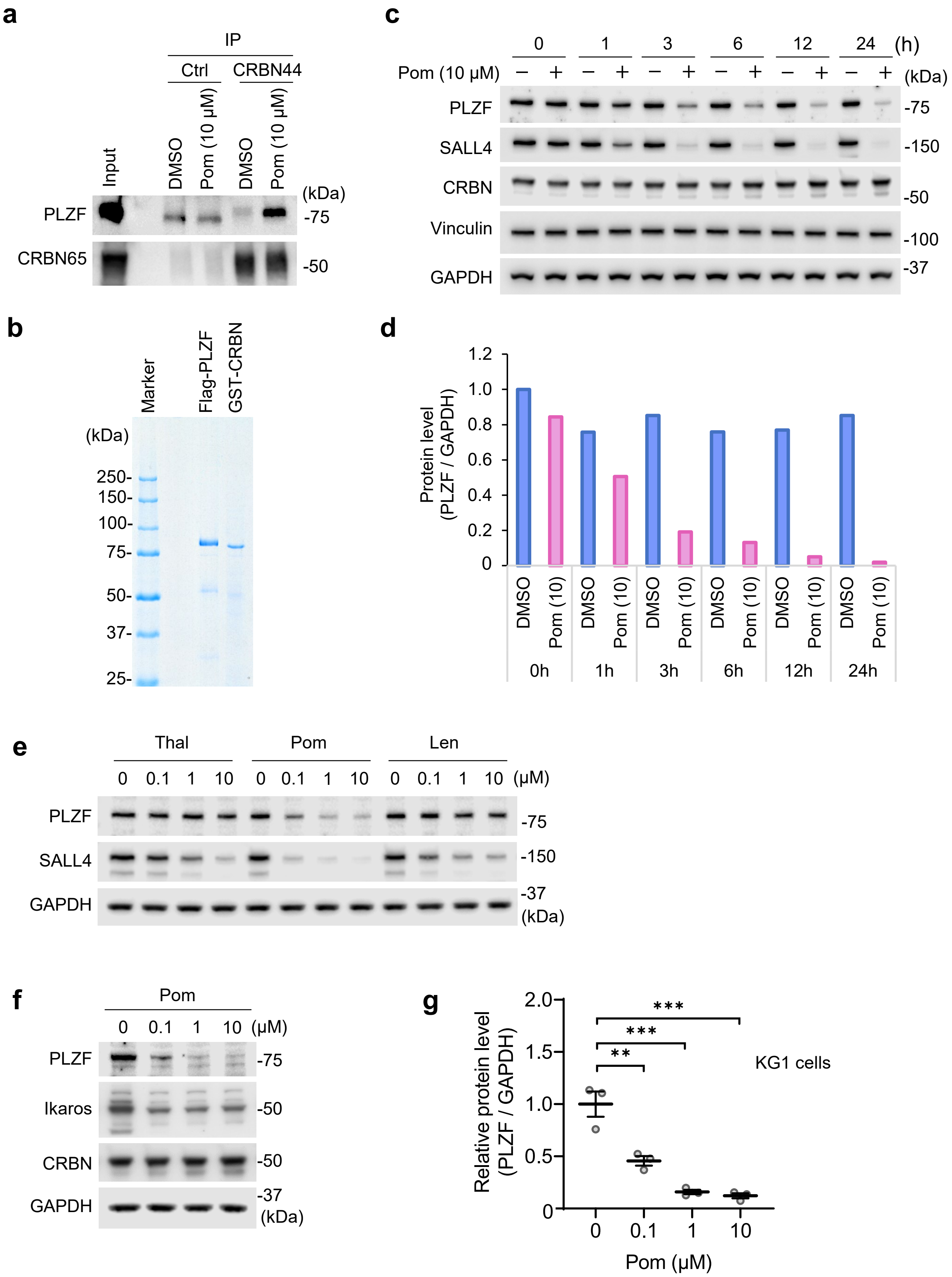
a
IP
Ctrl
CRBN44
Input
DMSO
Pom (10 μM)
DMSO
Pom (10 μM)
(kDa)
PLZF
-75
CRBN65
-50
b
Marker
Flag-PLZF
GST-CRBN
(kDa)
250-
150-
100-
75-
50-
37-
25-
c
0
1
3
6
12
24
(h)
Pom (10 μM)
– + – + – + – + – + – +
(kDa)
PLZF
-75
SALL4
-150
CRBN
-50
Vinculin
-100
GAPDH
-37
d
Protein level (PLZF / GAPDH)
1.2
1.0
0.8
0.6
0.4
0.2
0
DMSO
Pom (10)
0h
1h
3h
6h
12h
24h
e
Thal
Pom
Len
0 0.1 1 10 0 0.1 1 10 0 0.1 1 10 (μM)
PLZF
-75
SALL4
-150
GAPDH
-37
(kDa)
f
Pom
0 0.1 1 10 (μM)
PLZF
-75
Ikaros
-50
CRBN
-50
GAPDH
-37
(kDa)
g
Relative protein level (PLZF / GAPDH)
2.0
1.5
1.0
0.5
0
***
***
**
KG1 cells
0
0.1
1
10
Pom (μM)

Supplementary Figure 2. Further evidence supporting the findings in Figure 1.

a. Endogenous CRBN was immunoprecipitated from lt-NES cell extracts in the presence of DMSO or 10 μM pomalidomide, and the indicated samples were subjected to immunoblotting. **b**. Purified FLAG-tagged PLZF and GST-tagged CRBN were subjected to SDS-PAGE and CBB staining. **c.** Lt-NES cells were treated with pomalidomide for the indicated period and subjected to immunoblotting against the indicated antibodies. **d.** The immunoblot data shown in **c** were quantified, and relative intensities of PLZF/GAPDH were calculated and normalized to the control value (without pomalidomide). **e.** Sai2 cells were treated with the indicated drugs for 24 h and subjected to immunoblotting against the indicated antibodies. **f.** KG1 cells were treated with pomalidomide for 24 h and analyzed by immunoblotting against the indicated antibodies. **g.** The immunoblot data shown in **f** were quantified, and relative intensities of PLZF/GAPDH were calculated and normalized to the control value (without pomalidomide). Data are shown as mean ± SEM from three biologically independent samples. **, $p < 0.01$; ***, $p < 0.001$. Statistical significance was calculated with one-way Anova with multiple comparisons test. All experiments were conducted more than twice with similar results.

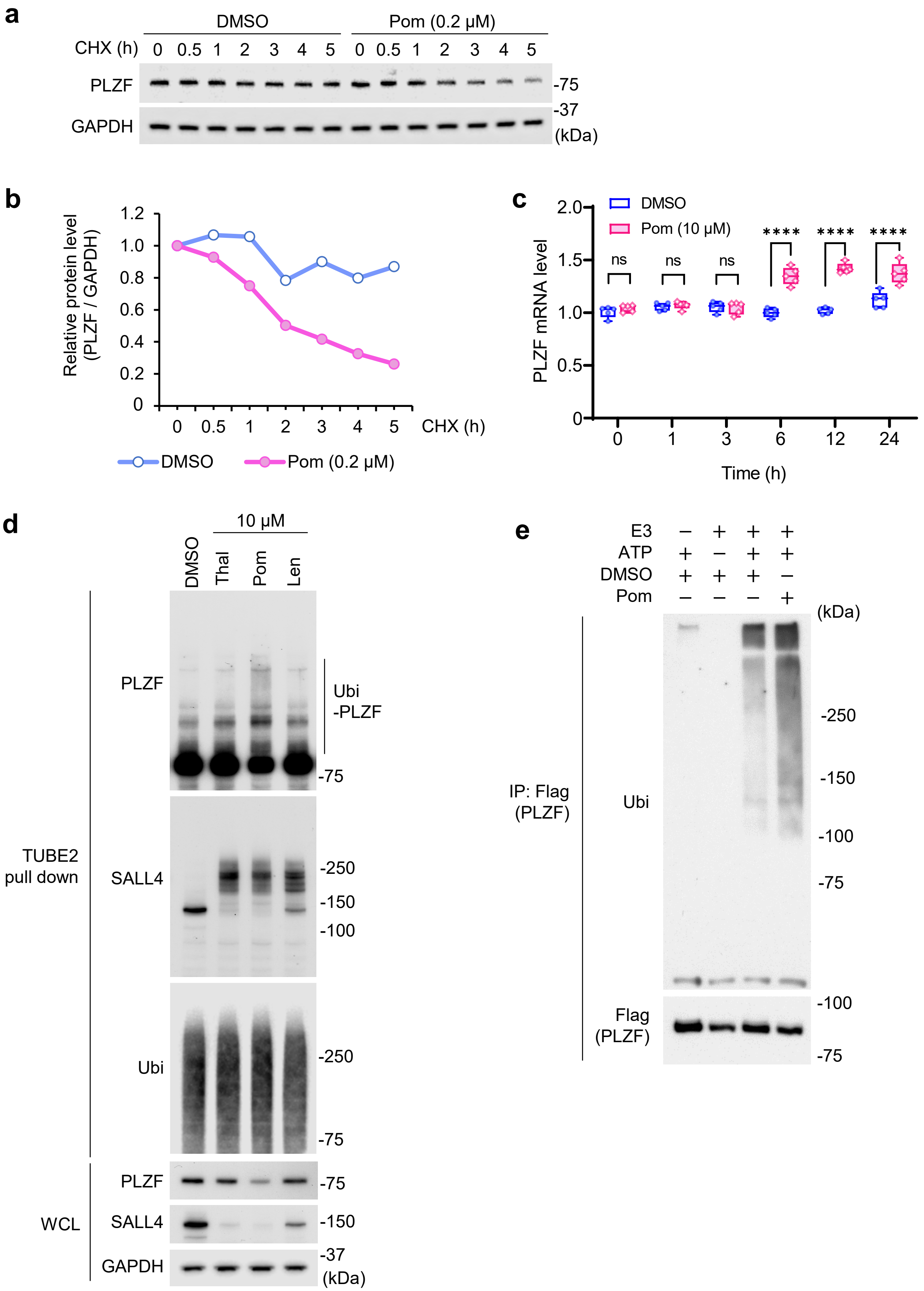

a
DMSO
Pom (0.2 μM)
CHX (h) 0 0.5 1 2 3 4 5 0 0.5 1 2 3 4 5
PLZF
GAPDH
-75
-37
(kDa)
b
Relative protein level (PLZF / GAPDH)
1.2
1.0
0.8
0.6
0.4
0.2
0
0 0.5 1 2 3 4 5 CHX (h)
DMSO
Pom (0.2 μM)
c
DMSO
Pom (10 μM)
PLZF mRNA level
2.0
1.5
1.0
0.5
0
ns
ns
ns
****
****
****
0 1 3 6 12 24
Time (h)
d
10 μM
DMSO
Thal
Pom
Len
TUBE2 pull down
PLZF
Ubi -PLZF
-75
SALL4
-250
-150
-100
Ubi
-250
-75
WCL
PLZF
-75
SALL4
-150
GAPDH
-37
(kDa)
e
E3 – + + +
ATP + – + +
DMSO + + + –
Pom – – – +
(kDa)
IP: Flag (PLZF)
Ubi
-250
-150
-100
-75
Flag (PLZF)
-100
-75

Supplementary Figure 3. Further evidence supporting the findings in Figure 2. a.

Immunoblot analysis of lt-NES cells treated with 50 µg/mL CHX and DMSO or 0.2 µM pomalidomide for the indicated periods. **b.** The immunoblot data shown in **a** were quantified, and relative intensities of PLZF/GAPDH were calculated (N=6). **c.** Lt-NES cells were treated with DMSO or pomalidomide for the indicated period and subjected to quantitative RT-PCR analysis. Relative mRNA amounts of *PLZF*/*GAPDH* were calculated and normalized to the control value (without pomalidomide). **d.** Lt-NES cells were treated with the indicated drugs or DMSO and MG132 for 6 h and subjected to pull-down with TUBE2 beads. Bound proteins were subjected to immunoblot analysis against the indicated antibodies. **e.** Affinity-purified Flag-tagged PLZF and $CRL4^{CRBN}$ (E3) were pre-incubated with DMSO or 100 µM pomalidomide (Pom) and then incubated with E1, E2, and ubiquitin in the presence or absence of ATP. Flag-PLZF was isolated using anti-Flag beads and visualized by immunoblot analysis using anti-Flag and anti-ubiquitin antibodies. WCL, whole cell lysate. Data are shown as mean ± SEM from six biologically independent samples from two independent experiments. ns, $p < 0.05$; ****, $p < 0.0001$. Statistical significance was calculated with two-way Anova with multiple comparisons test (**c**). All experiments were conducted more than twice with similar results.

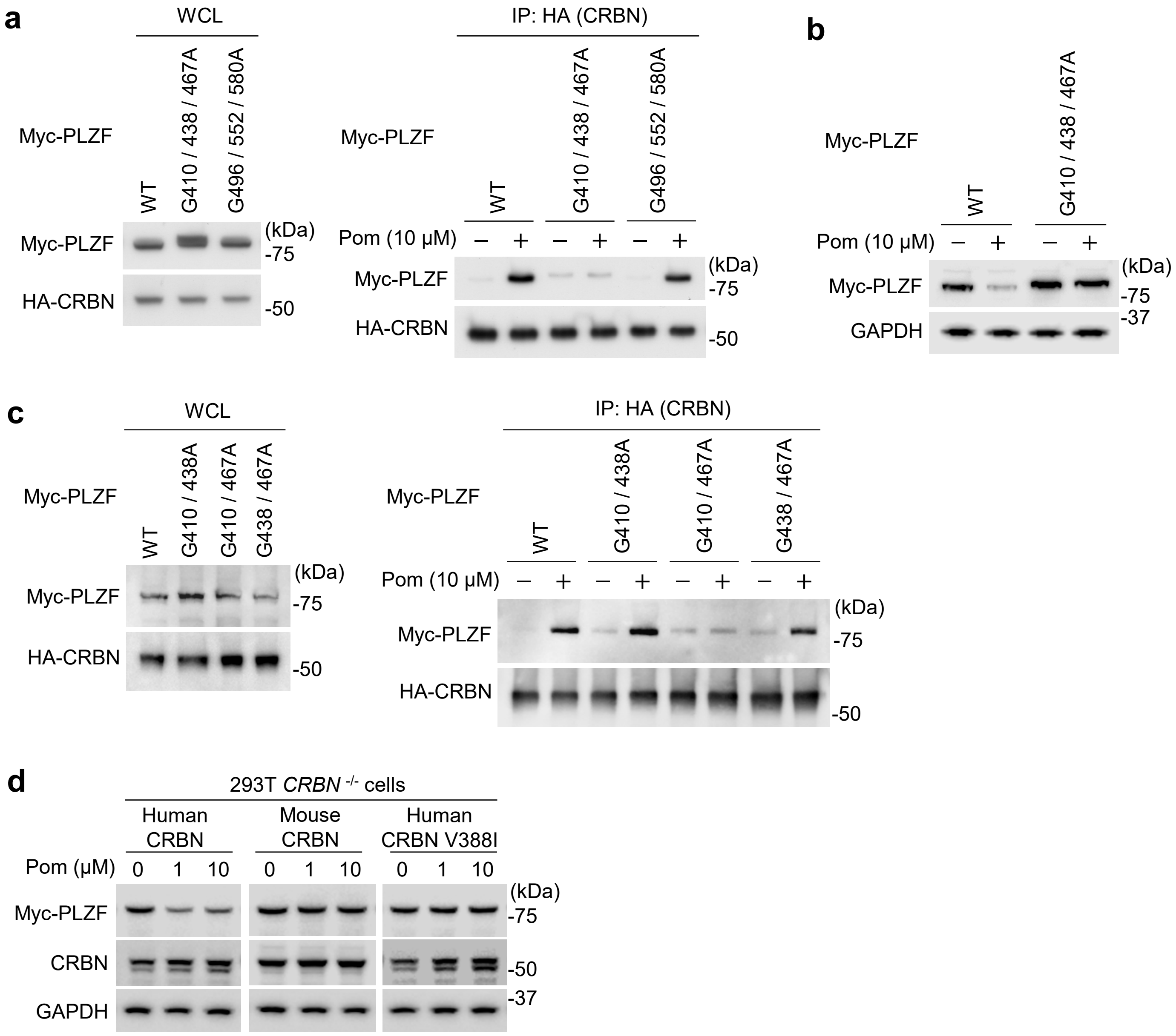

Supplementary Figure 4. Analysis of critical glycine residues of PLZF.

a. 293T cells expressing FH-CRBN were transfected with MF-tagged PLZF WT, $PLZF^{G410/438/467A}$, or $PLZF^{G496/552/580A}$ construct and subjected to immunoprecipitation using anti-HA agarose beads 48 h after transfection. After extensive washing, bound proteins were subjected to immunoblot analysis. **b.** 293T cells were transfected with MF-PLZF WT or $PLZF^{G410/438/467A}$ construct. Two days after transfection, pomalidomide was added where indicated, and the cells were further incubated for 24 h prior to harvesting for immunoblot analysis. **c.** 293T cells expressing FH-CRBN were transfected with MF-PLZF WT, $PLZF^{G410/438A}$, $PLZF^{G410/467A}$, or $PLZF^{G438/467A}$ construct and subjected to immunoprecipitation using anti-HA agarose beads 48 h after transfection. After extensive washing, bound proteins were subjected to immunoblot analysis. **d.** 293T cells lacking *CRBN* were transfected with MF-PLZF and FH-CRBN constructs as indicated. Next day, pomalidomide was added where indicated, and the cells were further incubated for 24 h prior to harvesting for immunoblot analysis. All experiments were conducted more than twice with similar results.

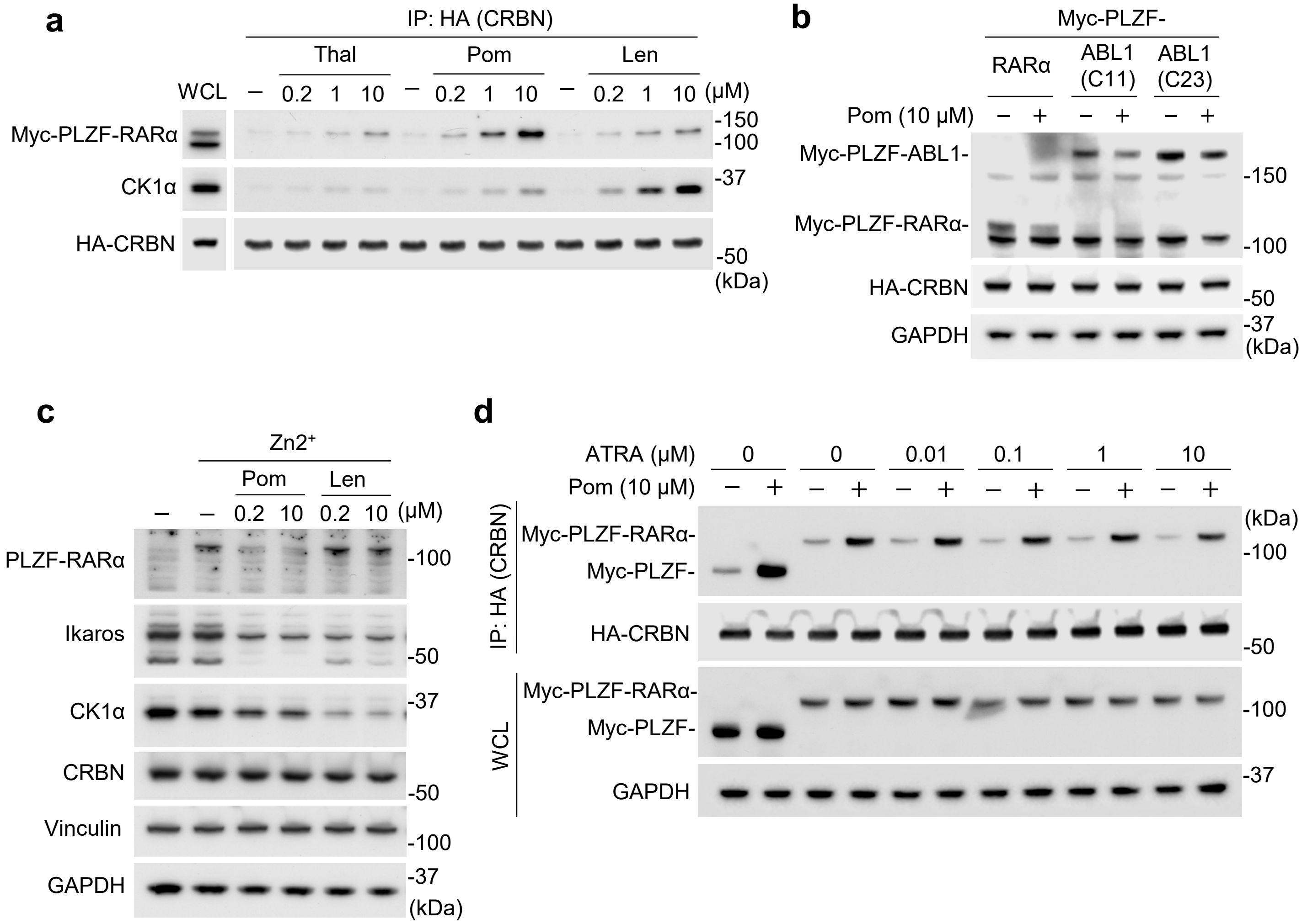

Supplementary Figure 5. Further biochemical analysis of PLZF-RARα and PLZF-ABL1.

a. 293T cells expressing FH-CRBN were transfected with Myc tagged-PLZF-RARα, incubated for 48 h, and then subjected to immunoprecipitation using anti-HA antibody in the presence of the indicated drugs. **b.** Myc-PLZF-RARα, Myc-PLZF-ABL1 (C11) and Myc-PLZF-ABL2 (C23) constructs were transfected into 293T cells expressing FH-CRBN. Next day, pomalidomide was added where indicated, and the cells were harvested 48 h after transfection and analyzed by immunoblotting against the indicated antibodies. **c.** B412 cells were treated with $ZnSO_4$ and the indicated drugs for 48 h. The drugs were replenished every 24 h. The cells were harvested and analyzed by immunoblotting against the indicated antibodies. **d.** 293T cells expressing FH-CRBN were transfected with Myc-PLZF-RARα construct and incubated for 48 h. The cells were harvested for immunoprecipitation using anti-HA antibody in the presence or absence of pomalidomide and ATRA at the indicated concentrations and analyzed by immunoblotting against the indicated antibodies. WCL, whole cell lysate. All experiments were conducted more than twice with similar results.

Source data for Figure 1a

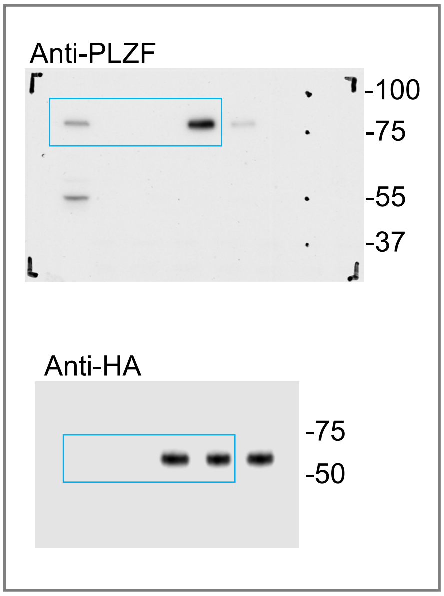

Source data for Figure 1b

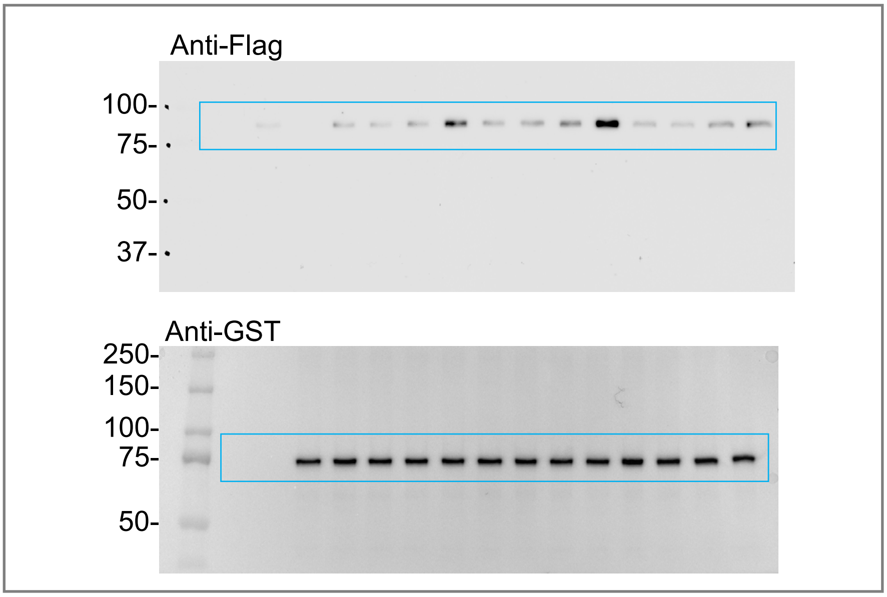

Source data for Figure 1c

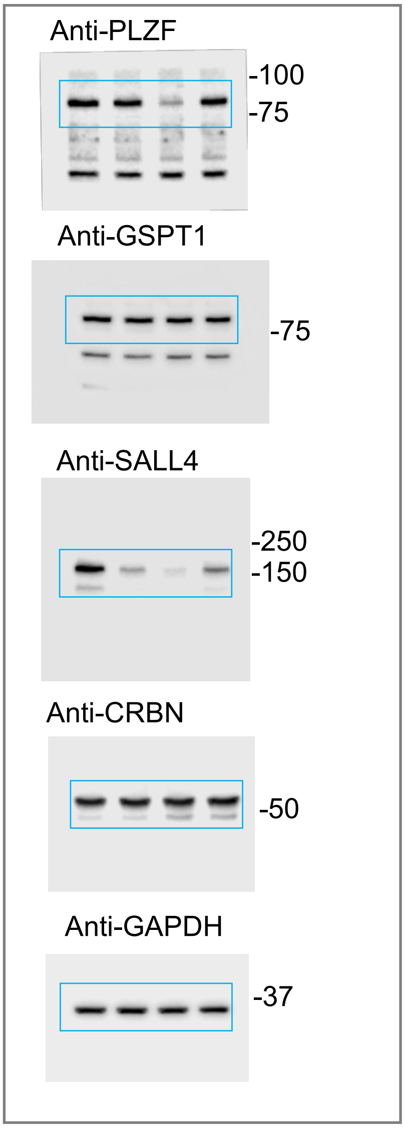

Source data for Figure 1d

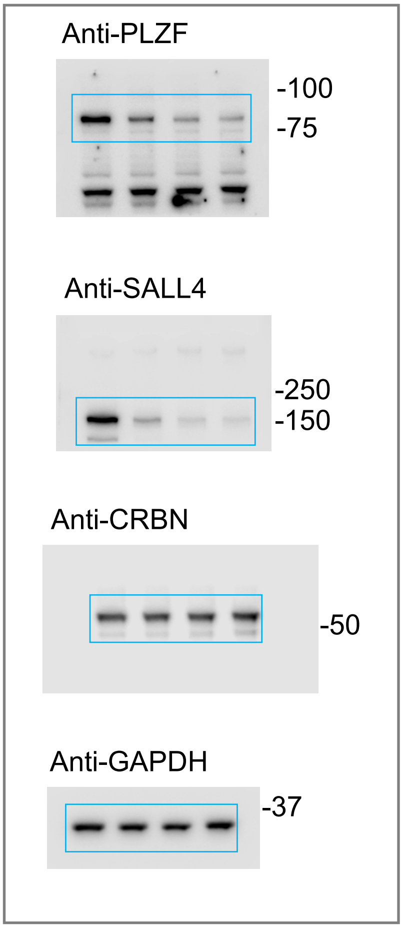

Source data for Figure 1h

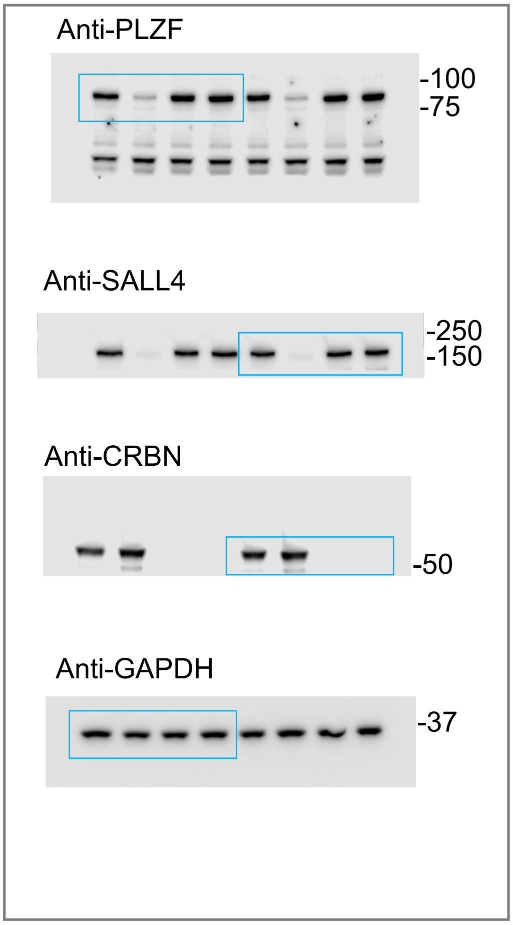

Supplementary Fig. 6. Full gel image related to the indicated figures. **(continued on next page)**

Source data for Figure 2a

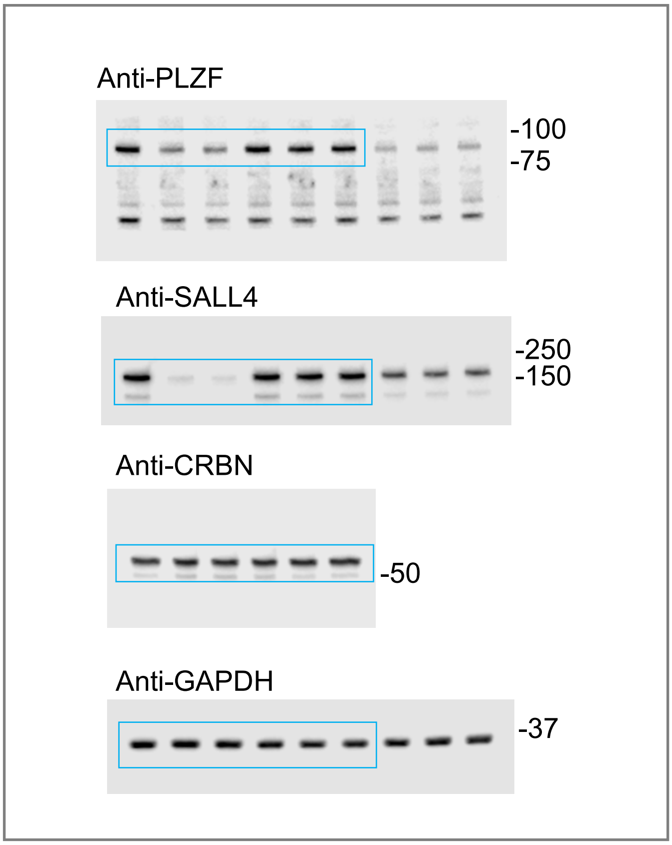

Source data for Figure 2b

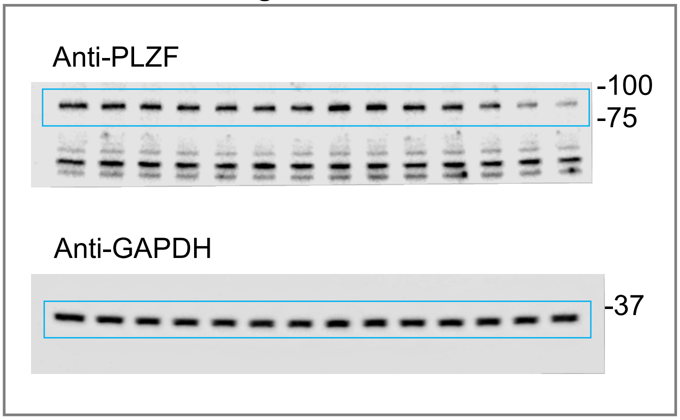

Source data for Figure 2d

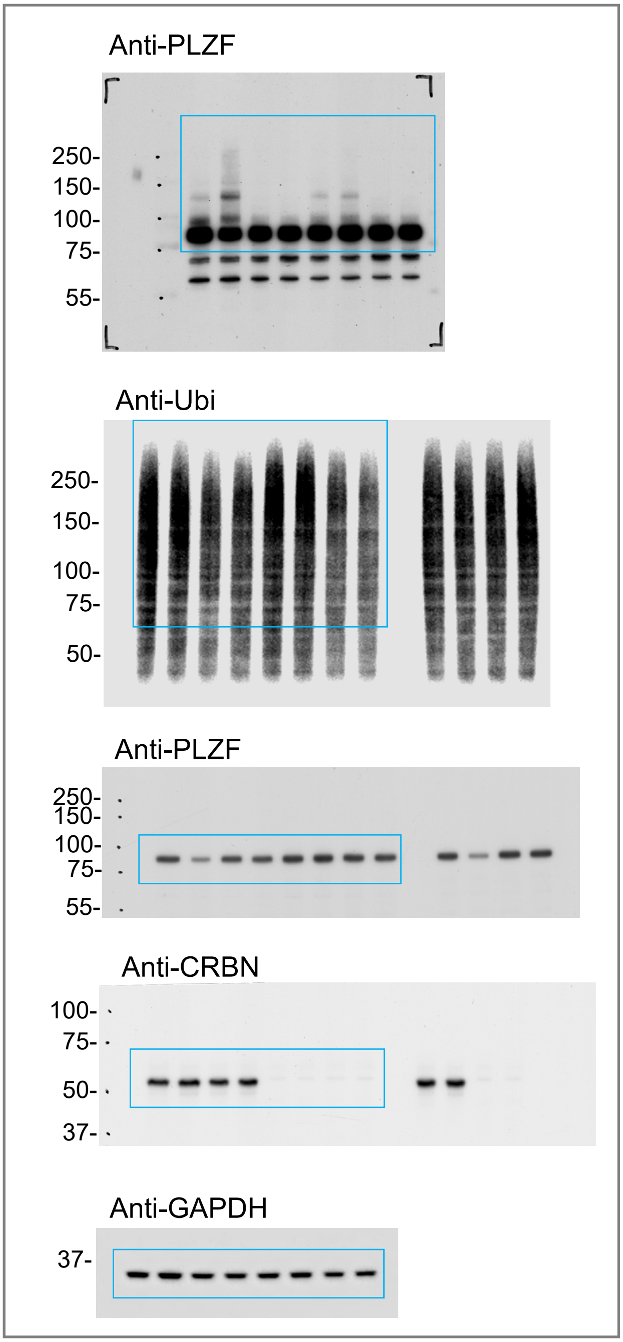

Supplementary Fig. 6. Full gel image related to the indicated figures. **(continued on next page)**

Source data for Figure 3c

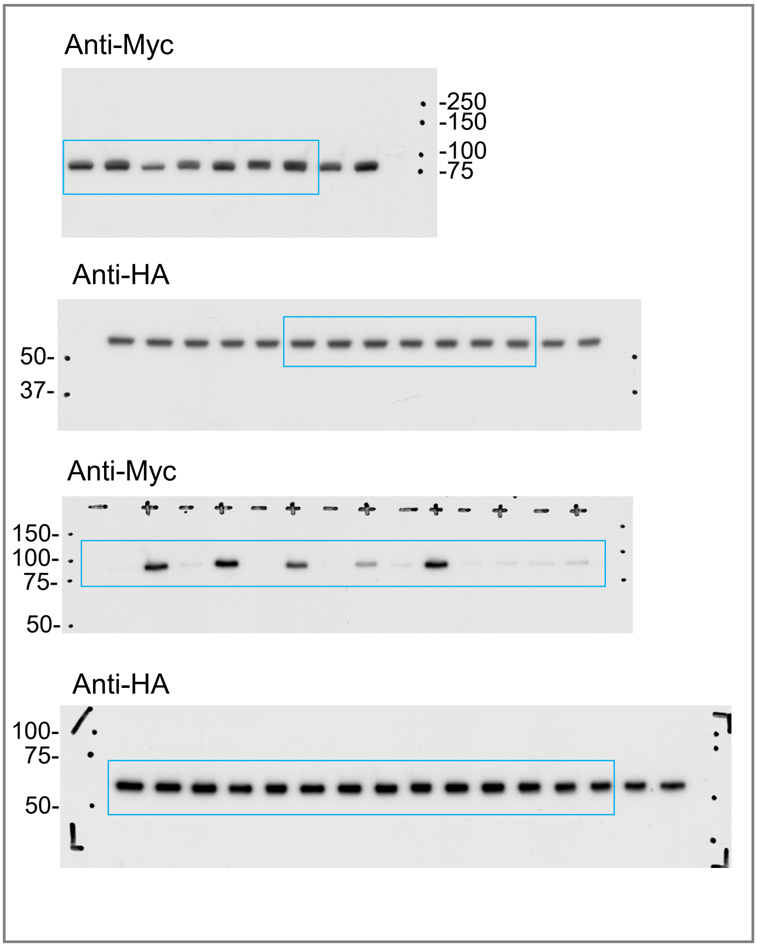

Source data for Figure 3e

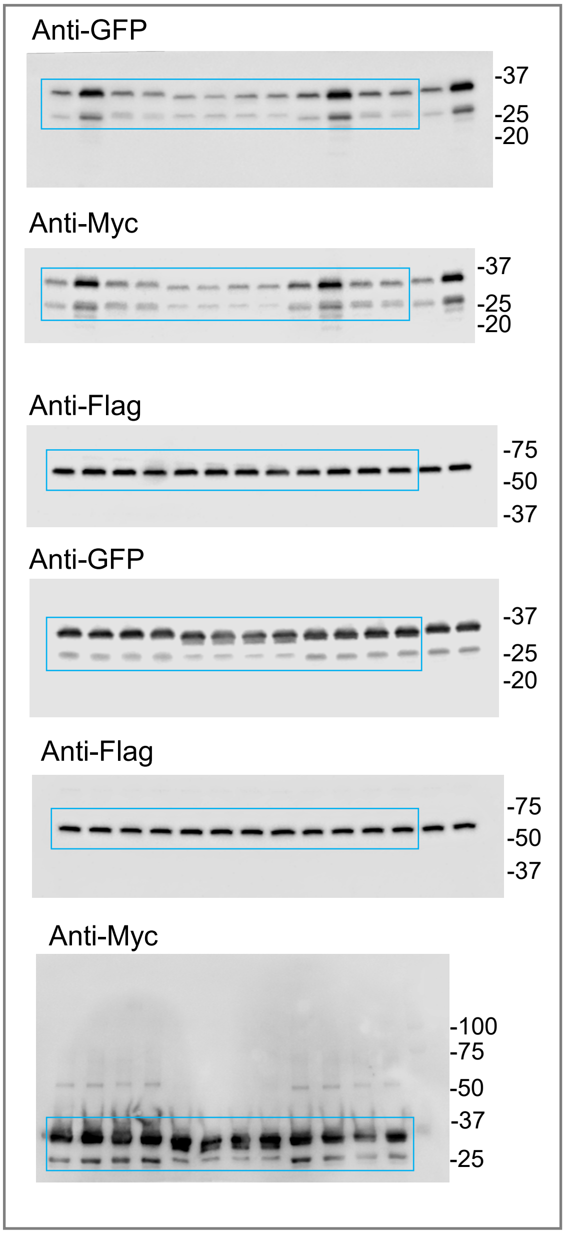

Supplementary Fig. 6. Full gel image related to the indicated figures. **(continued on next page)**

Source data for Figure 4b

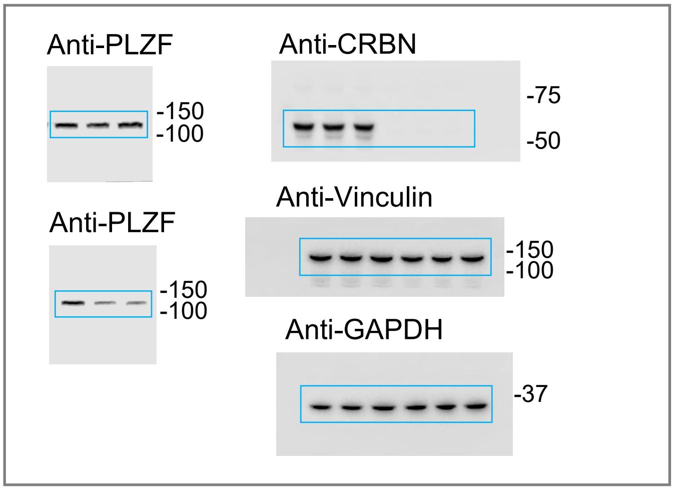

Source data for Figure 4c

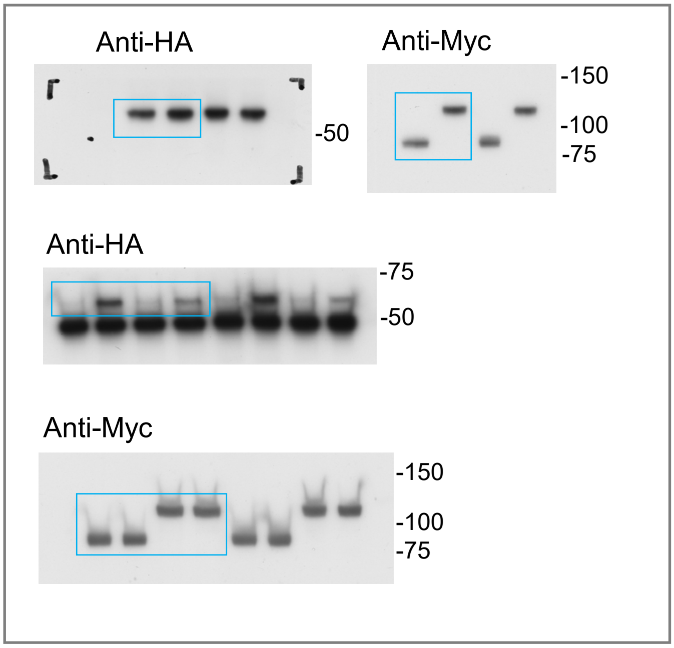

Source data for Figure 4d

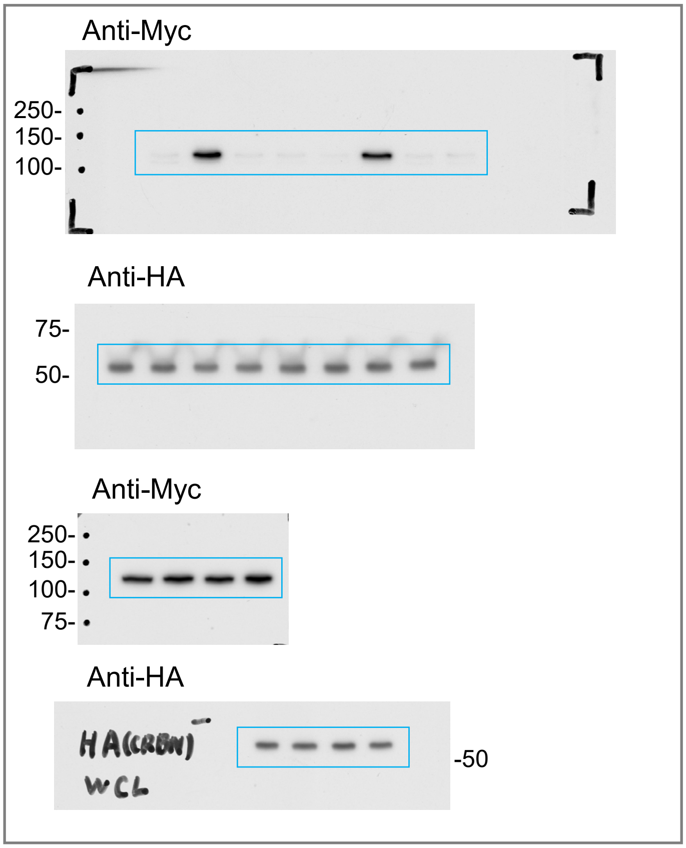

Source data for Figure 4e

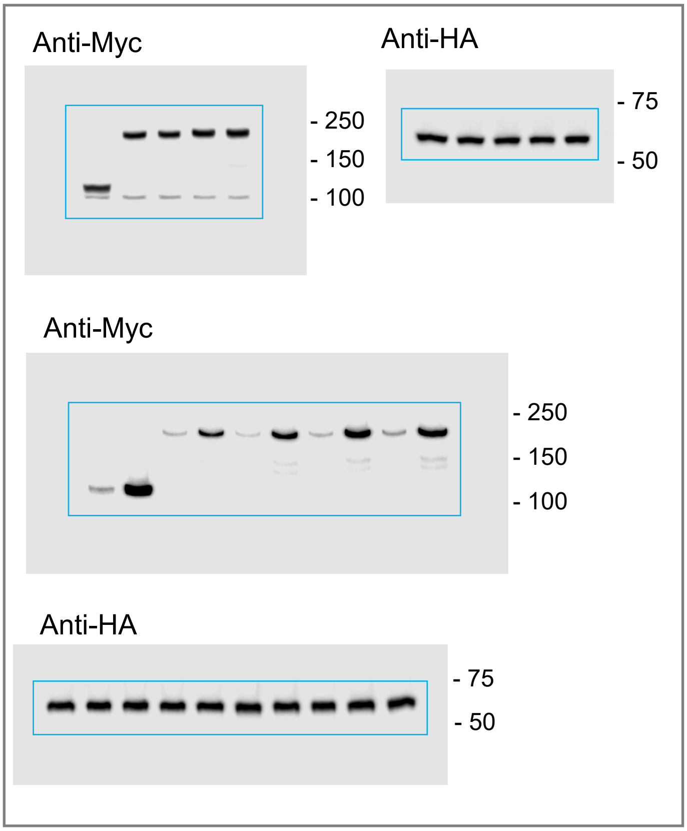

Supplementary Fig. 6. Full gel image related to the indicated figures. **(continued on next page)**

Source data for Figure 5a

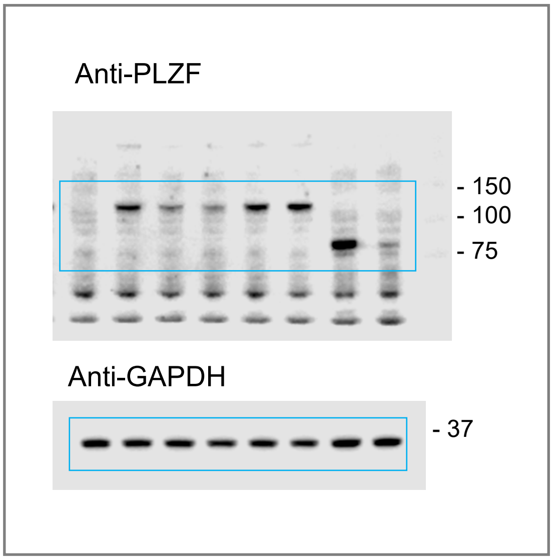

Source data for Figure 5c

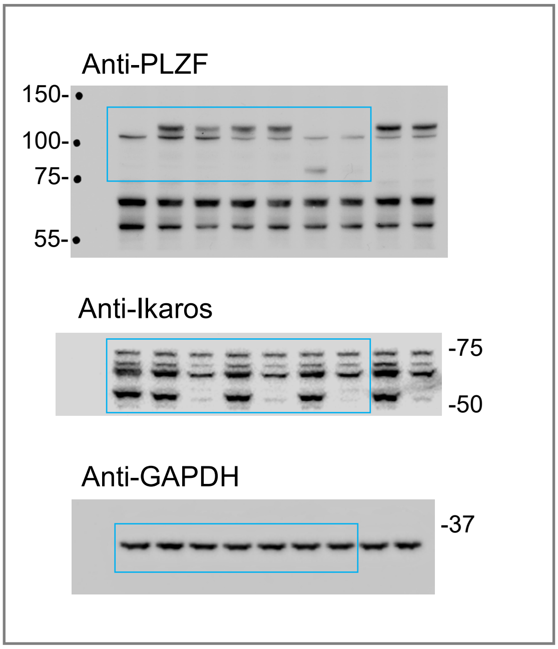

Supplementary Fig. 6. Full gel image related to the indicated figures. (continued on next page)

Source data for Supplementary Figure 2b

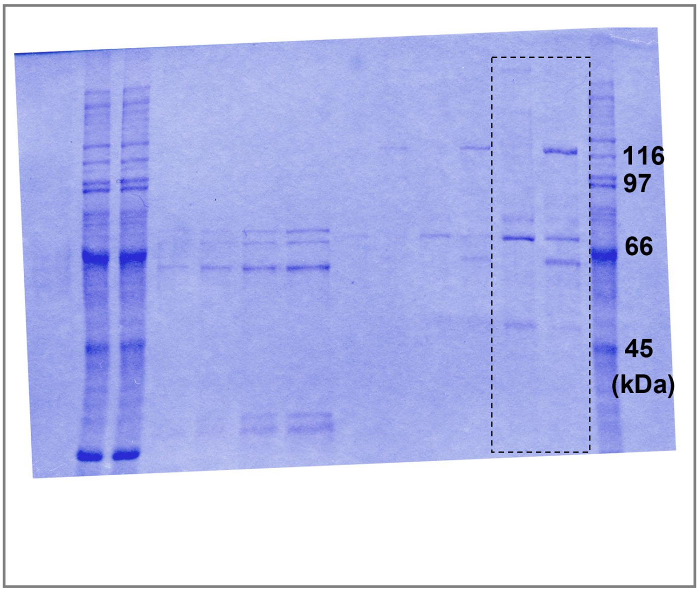

Source data for Supplementary Figure 2c

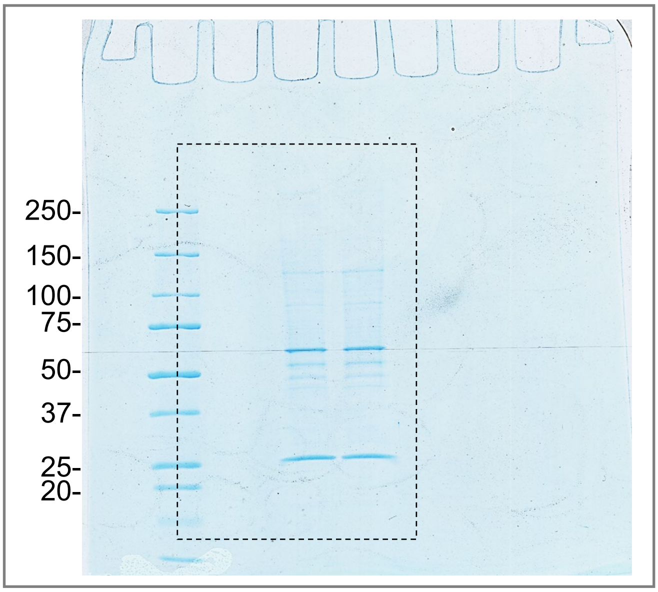

Supplementary Fig. 6. Full gel image related to the indicated figures. **(continued on next page)**

Source data for Supplementary Figure 2a

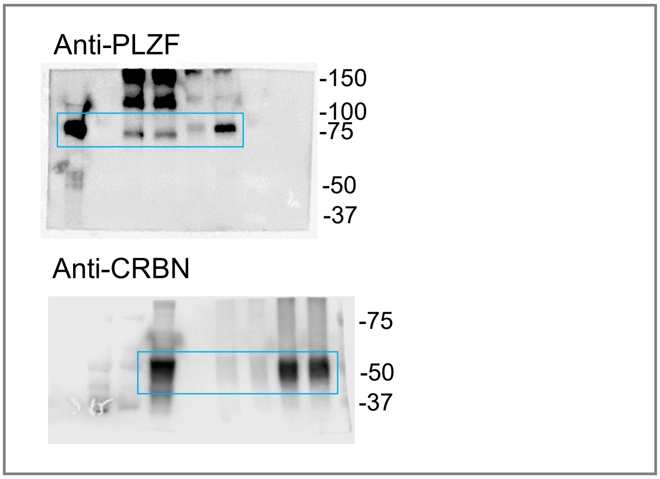

Source data for Supplementary Figure 2b

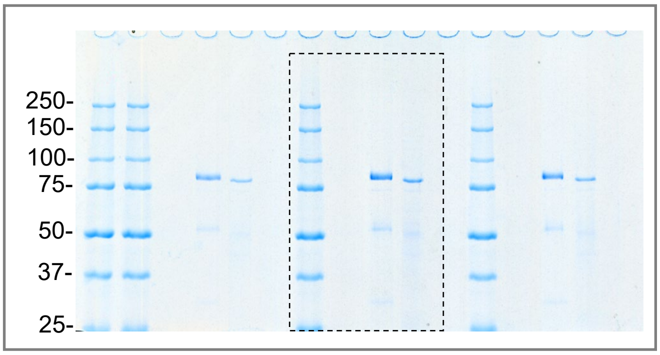

Source data for Supplementary Figure 2c

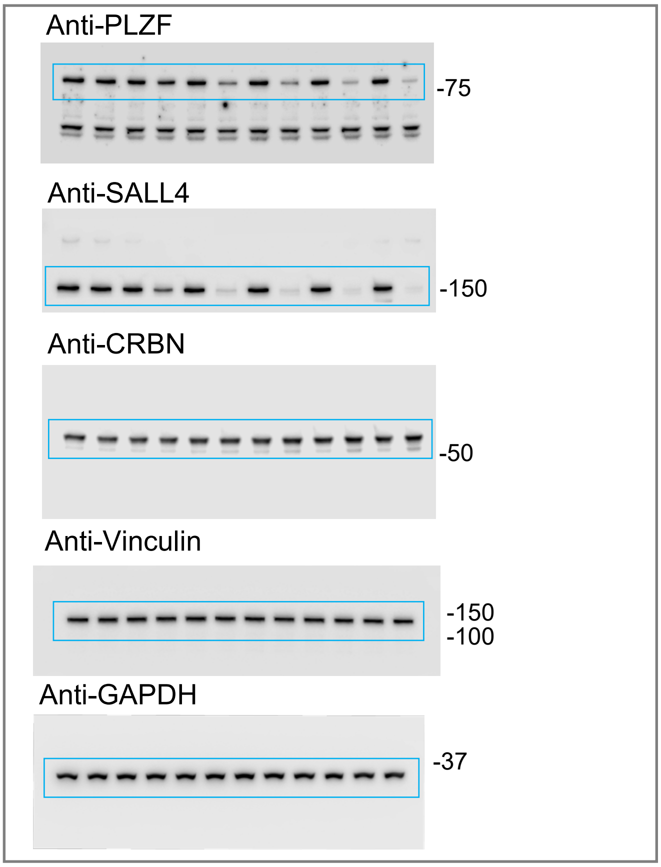

Source data for Supplementary Figure 2e

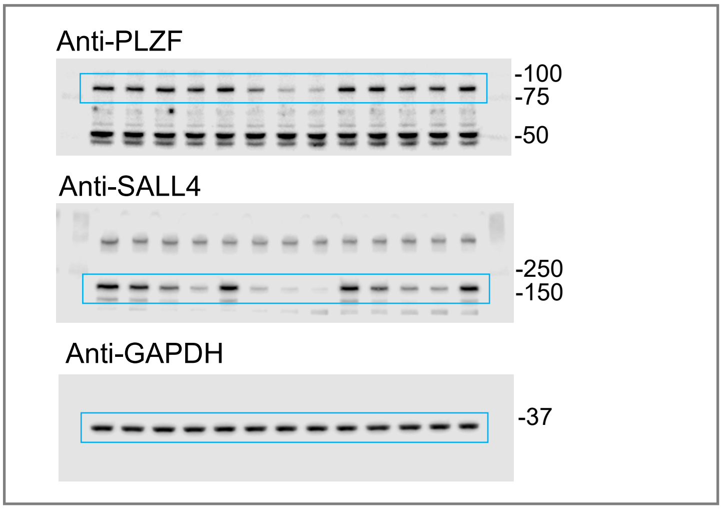

Source data for Supplementary Figure 2f

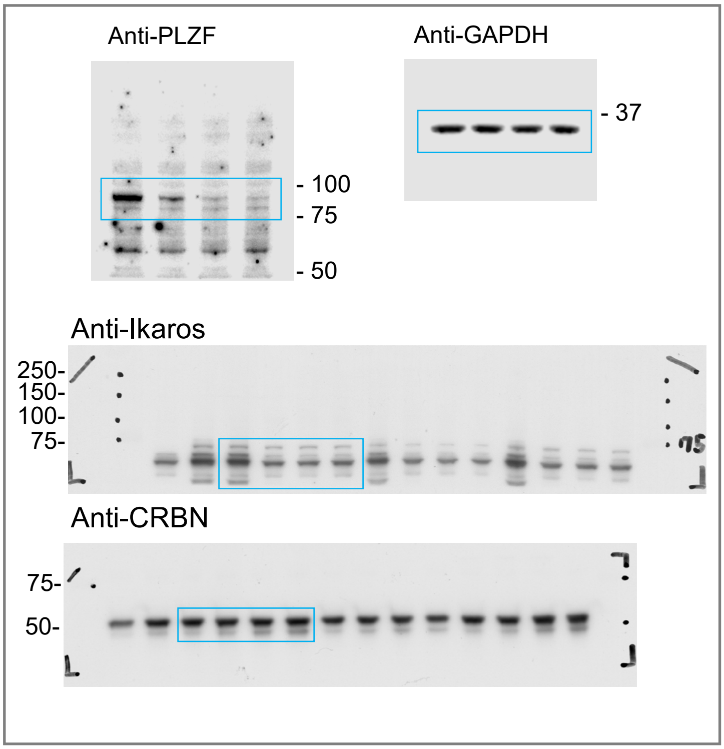

Supplementary Fig. 6. Full gel image related to the indicated figures. **(continued on next page)**

Source data for Supplementary Figure 3a

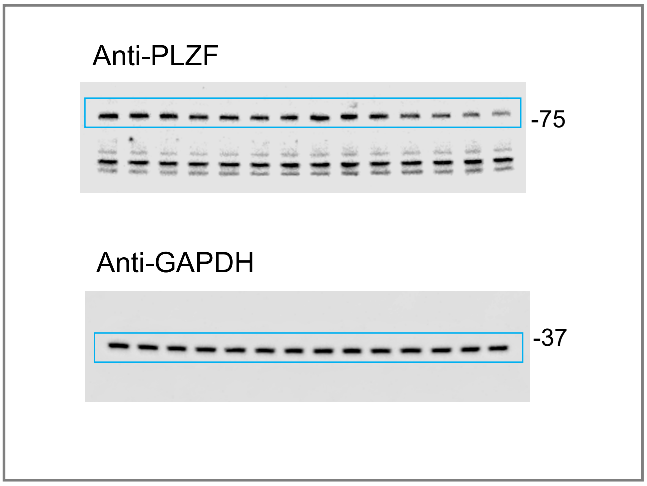

Source data for supplementary Figure 3e

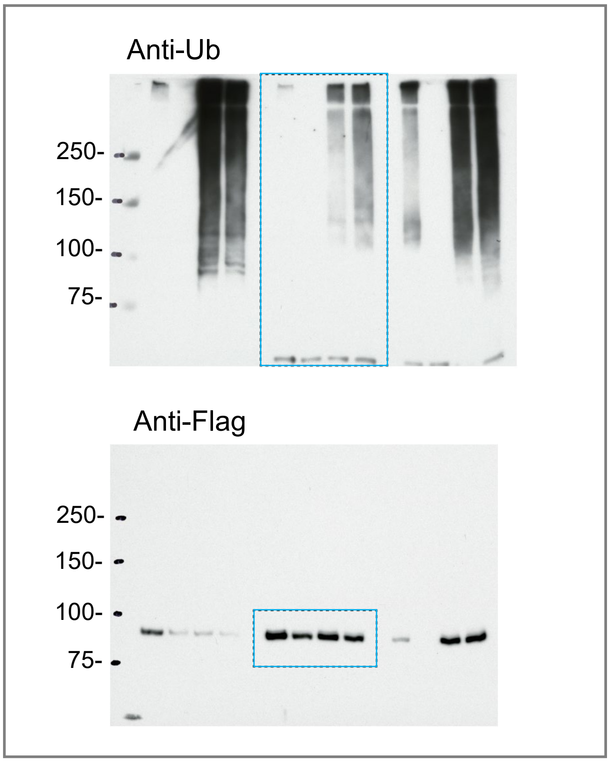

Source data for supplementary Figure 3d

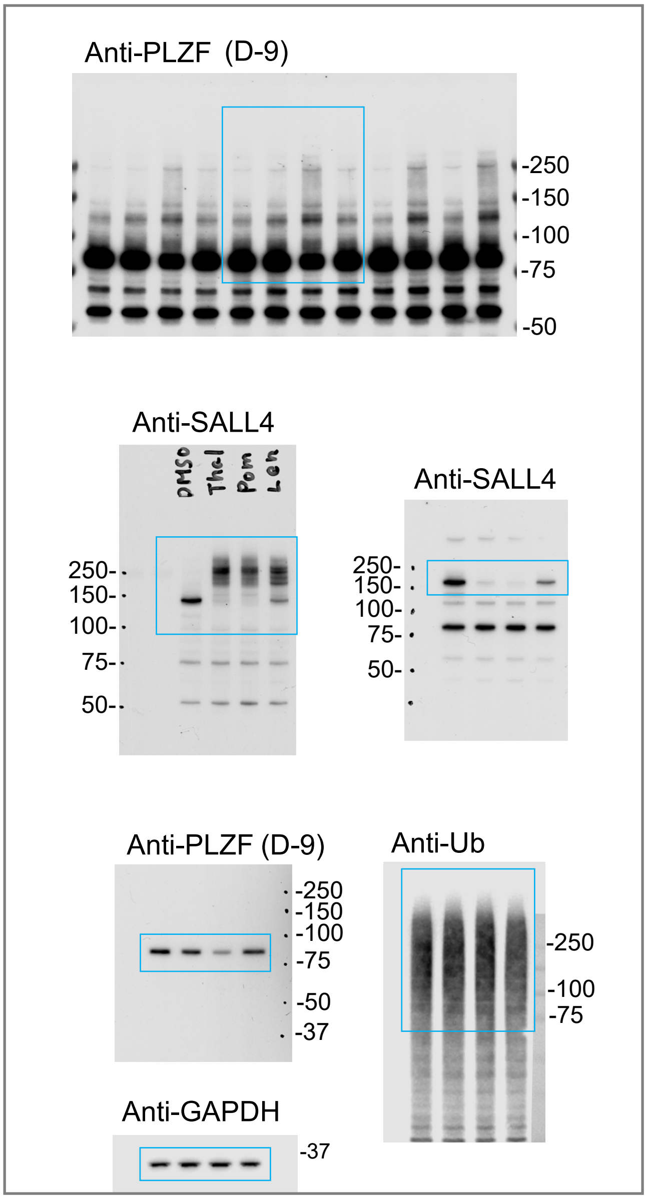

Supplementary Fig. 6. Full gel image related to the indicated figures. **(continued on next page)**

Source data for Supplementary Figure 4a

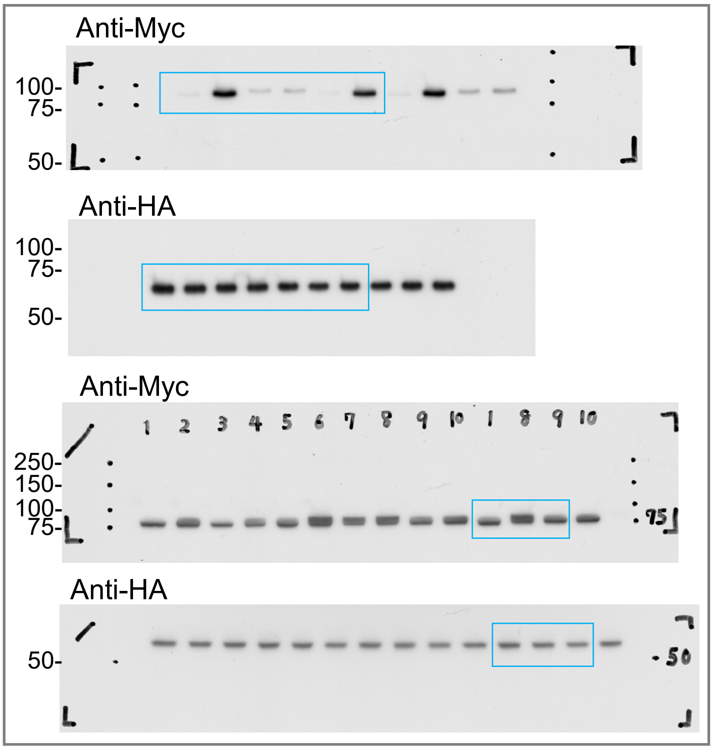

Source data for Supplementary Figure 4d

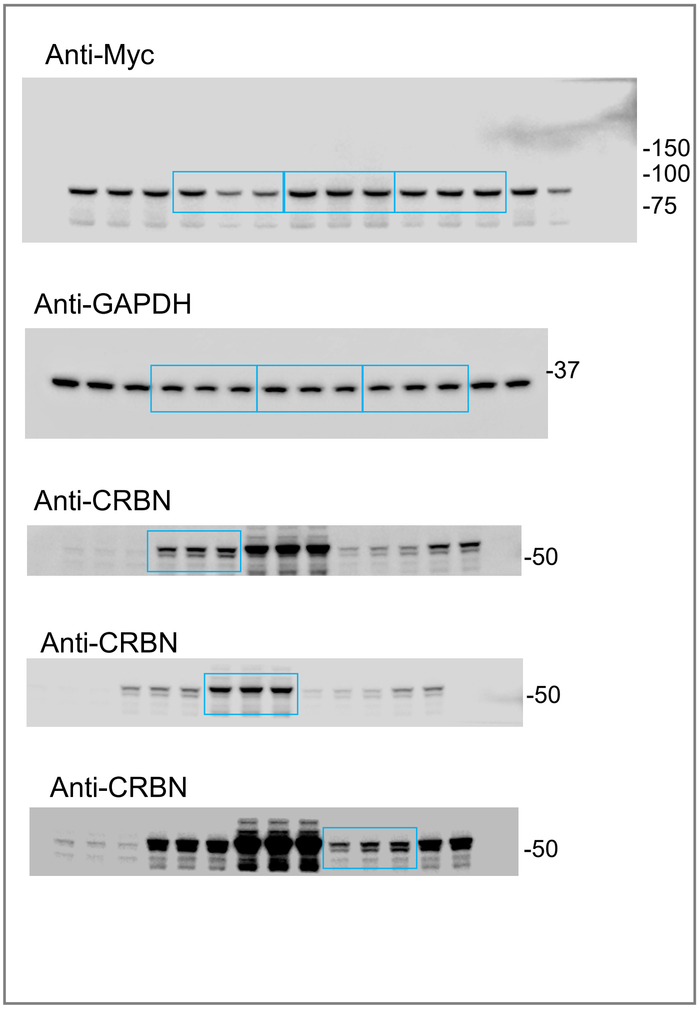

Source data for Supplementary Figure 4b

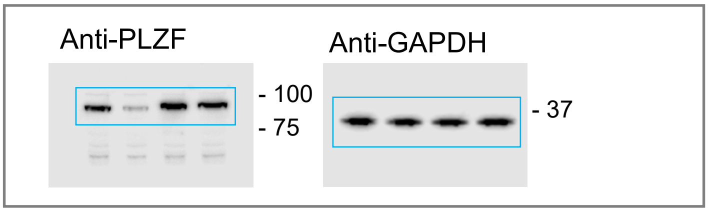

Source data for Supplementary Figure 4c

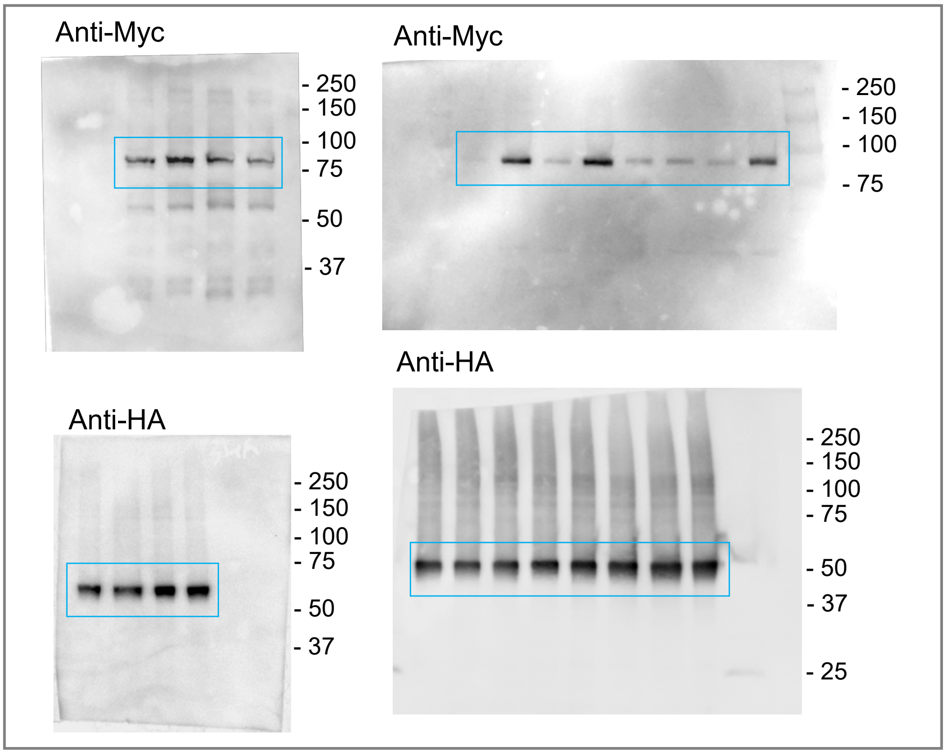

Supplementary Fig. 6. Full gel image related to the indicated figures. **(continued on next page)**

Source data for Supplementary Figure 5a

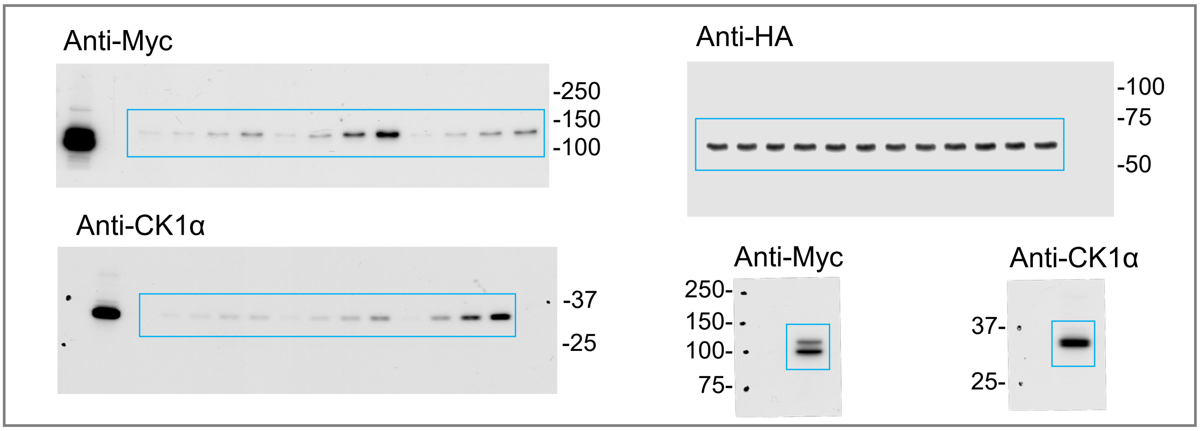

Source data for Supplementary Figure 5b

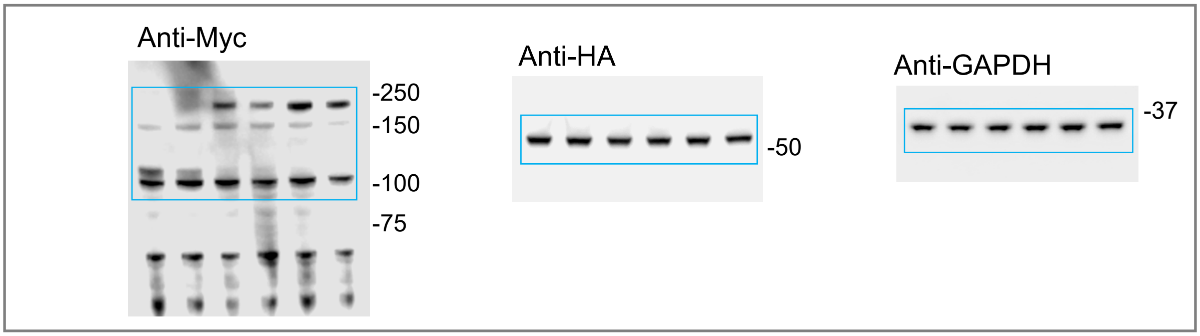

Source data for Supplementary Figure 5c

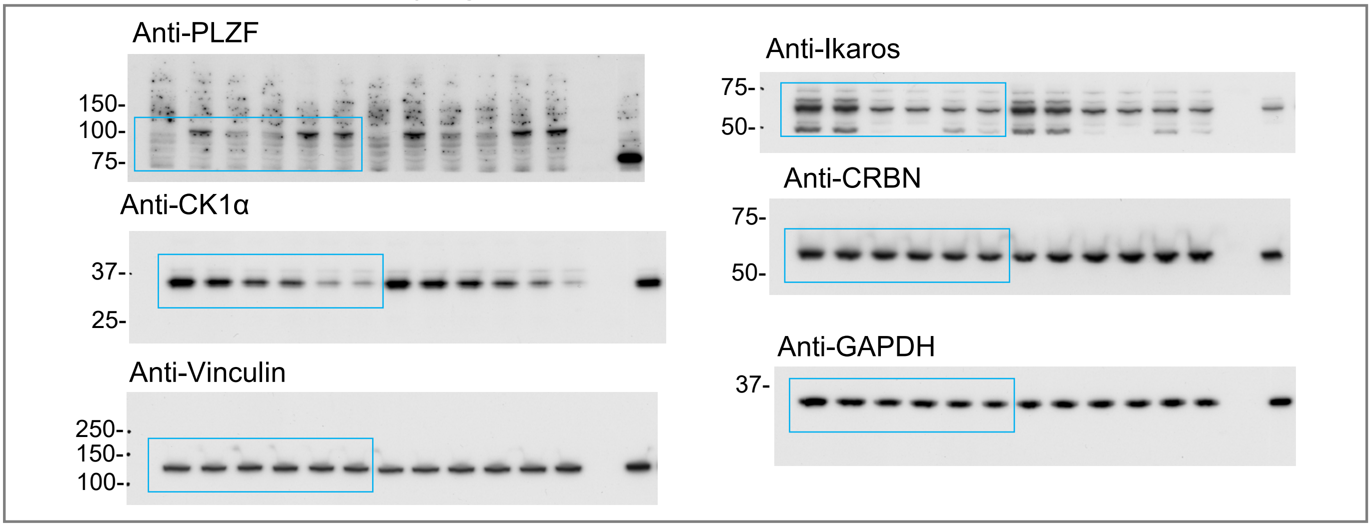

Source data for Supplementary Figure 5d

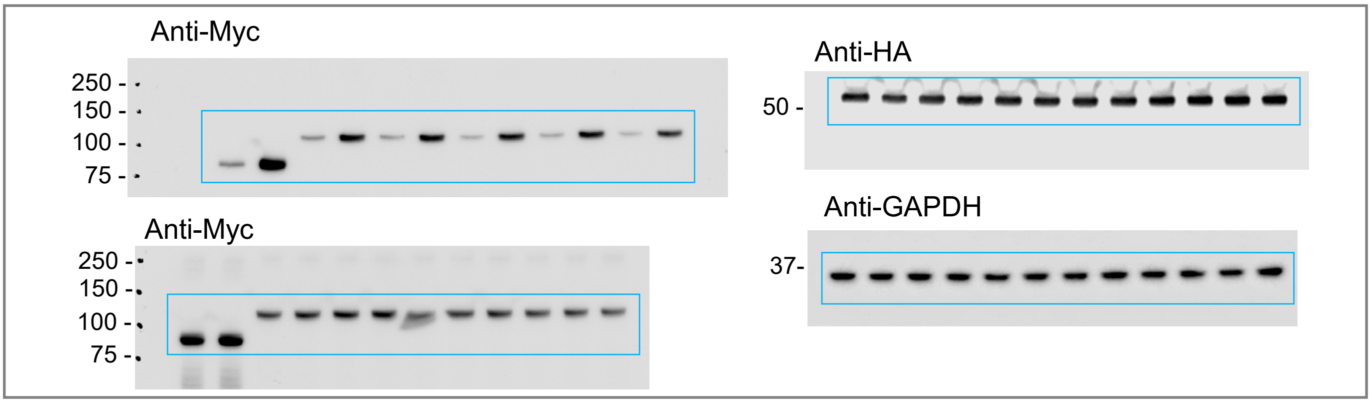

Supplementary Fig. 6. Full gel image related to the indicated figures.
