# Supplementary material for: PLZF and its fusion proteins are pomalidomide-dependent CRBN neosubstrates
Source: Commun Biol. 2021 Nov 11;4:1277. doi: 10.1038/s42003-021-02801-y (PMC8586336; doi:10.1038/s42003-021-02801-y)
Supplement: Supplementary file 4 — Reporting Summary [file 42003_2021_2801_MOESM4_ESM.pdf]

nature research

Corresponding author(s): Hiroshi Handa and Takumi Ito

Last updated by author(s): Sep 13, 2021

Reporting Summary

Nature Research wishes to improve the reproducibility of the work that we publish. This form provides structure for consistency and transparency in reporting. For further information on Nature Research policies, see our Editorial Policies and the Editorial Policy Checklist.

Statistics

For all statistical analyses, confirm that the following items are present in the figure legend, table legend, main text, or Methods section.

n/a	Confirmed	
☐	☒	The exact sample size (*n*) for each experimental group/condition, given as a discrete number and unit of measurement
☐	☒	A statement on whether measurements were taken from distinct samples or whether the same sample was measured repeatedly
☐	☒	The statistical test(s) used AND whether they are one- or two-sided<br>*Only common tests should be described solely by name; describe more complex techniques in the Methods section.*
☒	☐	A description of all covariates tested
☒	☐	A description of any assumptions or corrections, such as tests of normality and adjustment for multiple comparisons
☐	☒	A full description of the statistical parameters including central tendency (e.g. means) or other basic estimates (e.g. regression coefficient) AND variation (e.g. standard deviation) or associated estimates of uncertainty (e.g. confidence intervals)
☐	☒	For null hypothesis testing, the test statistic (e.g. *F*, *t*, *r*) with confidence intervals, effect sizes, degrees of freedom and *P* value noted<br>*Give P values as exact values whenever suitable.*
☒	☐	For Bayesian analysis, information on the choice of priors and Markov chain Monte Carlo settings
☒	☐	For hierarchical and complex designs, identification of the appropriate level for tests and full reporting of outcomes
☒	☐	Estimates of effect sizes (e.g. Cohen's *d*, Pearson's *r*), indicating how they were calculated

Our web collection on statistics for biologists contains articles on many of the points above.

Software and code

Policy information about availability of computer code

Data collection	Mass spectrometry: Ultimate 3000 RSLCnano liquid chromatography (Thermo Fisher Scientific), Q Exactive Orbitrap mass spectrometer (Thermo Fisher Scientific)<br>qRT-PCR analysis: CFX96 Real-Time System (Bio-Rad)<br>Immunocytochemistry: EVOS Imaging System (Thermo Fisher Scientific)<br>Proliferation analysis: iMark microplate absorbance reader (Bio-Rad)
Data analysis	Calculation and statistics: Microsoft Excel (Microsoft), Prism 8 (GraphPad Software)<br>Mass spectrometry data analysis: Proteome Discoverer (ver. 2.2) (Thermo Fisher Scientific), Mascot (Matrix Science)<br>Semi-quantitative analysis of WB: Fusion FX (VILBER)<br>Semi-quantitative analysis of ICC: ImageJ 1.51s (NIH)<br>qRT-PCR analysis: CFX manager (Bio-Rad)<br>Data and images processing: Photoshop CC2021 (Adobe), Illustrator CC2021 (Adobe), Microsoft PowerPoint (Microsoft)

For manuscripts utilizing custom algorithms or software that are central to the research but not yet described in published literature, software must be made available to editors and reviewers. We strongly encourage code deposition in a community repository (e.g. GitHub). See the Nature Research guidelines for submitting code & software for further information.

Data

Policy information about availability of data

All manuscripts must include a data availability statement. This statement should provide the following information, where applicable:

- Accession codes, unique identifiers, or web links for publicly available datasets
- A list of figures that have associated raw data
- A description of any restrictions on data availability

The data that support the findings of this study are available from the corresponding author upon reasonable request.
The mass spectrometry proteomics data are deposited in the Japan Proteome Standard Repository/Database (jPOST), a member of the ProteomeXchange consortium. The accession numbers are PXD023928 for ProteomeXchange and JPST001075 for jPOST.

Field-specific reporting

Please select the one below that is the best fit for your research. If you are not sure, read the appropriate sections before making your selection.

☒ Life sciences ☐ Behavioural & social sciences ☐ Ecological, evolutionary & environmental sciences

For a reference copy of the document with all sections, see nature.com/documents/nr-reporting-summary-flat.pdf

Life sciences study design

All studies must disclose on these points even when the disclosure is negative.

Sample size	We did not compute statistical analyses to predetermine sample sizes. Sample sizes were chosen based on our previous study and similar studies by others. All analyses except MS spectrometry were conducted twice or more independently, each experimental repeat yielding similar results.
Data exclusions	No data were excluded.
Replication	We have reproduced all our results except MS data in a minimum of two independent experimental repeats. Experimental findings have also been reproduced independently by co-authors of the paper using the same experimental conditions.
Randomization	Randomization was not performed. No human participants or animal models were involved in this study.
Blinding	Partial blinding was employed for proliferation assay shown in Fig. 6a and 6c. Blinding was not conducted for other experiments. We conducted automated sample collection and data quantitative data analysis without human intervention where possible.

Reporting for specific materials, systems and methods

We require information from authors about some types of materials, experimental systems and methods used in many studies. Here, indicate whether each material, system or method listed is relevant to your study. If you are not sure if a list item applies to your research, read the appropriate section before selecting a response.

Materials & experimental systems

n/a	Involved in the study	
☐	☒	Antibodies
☐	☒	Eukaryotic cell lines
☒	☐	Palaeontology and archaeology
☒	☐	Animals and other organisms
☒	☐	Human research participants
☒	☐	Clinical data
☒	☐	Dual use research of concern

Methods

n/a	Involved in the study	
☒	☐	ChIP-seq
☒	☐	Flow cytometry
☒	☐	MRI-based neuroimaging

Antibodies

Antibodies used	For immunoblotting, rabbit anti-human CRBN65 mAb (Celgene, San Diego, CA, 1:10000 dilution, Lot# CGN-6-4-5), mouse anti-PLZF mAb (39987, Active Motif, 1:1000 dilution, Lot#05313002), mouse anti-PLZF mAb (sc-28319, Santa Cruz, 1:1000 dilution, Lot#C0618), mouse anti-SALL4 mAb (ab57577, Abcam, 1:1000 dilution, Lot#GR3198290-2), mouse anti-Vinculin mAb (ab18058, Abcam, 1:1000 dilution, Lot#GR207163-1), mouse anti-HA11 mAB (901503, BioLegend, 1:1000 dilution, Lot#b207273), mouse anti-Flag M2 mAB (F1804, SIGMA, 1:1000 dilution, Lot#SLBS3530V), rabbit anti-Myc tag (ab9106, Abcam, 1:1000 dilution), rabbit anti-GSPT1 (ab49878, Abcam, 1:1000 dilution, Lot#GR274469-4), rabbit anti-CK1α mAB (ab108296, Abcam, 1:1000 dilution, Lot#GR53415-9), rabbit anti-Ikaros mAB (ab26083, Abcam, 1:1000 dilution, Lot#GR250435-1), rabbit anti-GFP (ab290, Abcam, 1:1000 dilution, Lot# GR 3184825-1) were used as primary antibodies. Anti-mouse IgG, HRP-linked Antibody (#7076, Cell Signaling, 1:10000 dilution, Lot#25),

	anti-rabbit IgG, HRP-linked Antibody (#7074, Cell Signaling, 1:10000 dilution, Lot#33) were used as secondary antibodies. Mouse anti-GAPDH-mAb-HRP-DirecT (M171-7, MBL, 1:5000 dilution, Lot#006), mouse anti-Myc-tag mAb-HRP-DirecT (M192-7, MBL, 1:2000 dilution, Lot#006), mouse anti-Ub (P4D1)-mAb-HRP (sc-8017, Santa Cruz, 1:1000 dilution, Lot#A0919), anti-Ub mAb (FK2, ENZO, 1:1000 dilution, Lot#07281715), rabbit anti-GST-tag pAb-HRP-DirecT (PM013-7, MBL, 1:1000 dilution, Lot#005) were also used. For immunostaining, mouse anti-PLZF mAb (39987, Active Motif, 1:200 dilution, Lot#05313002), rabbit anti-SOX2 (ab92494, Abcam, 1:200 dilution, Lot#GR30587-10), Alexa Fluor 488 goat anti-mouse IgG (A11029, Invitrogen, 1:500 dilution, Lot#1911843), Alexa Fluor 555 goat anti-rabbit IgG (A21429, Invitrogen, 1:500 dilution, Lot#1911843) were used as secondary antibodies. For immunoprecipitation, mouse anti-human CRBN44 antibody, generated against 1-18 of human CRBN, was used.
Validation	All the antibodies used in the study, except for anti-human CRBN65 mAb and CRBN44 mAb, are commercially available. Anti-CRBN65 was used for several publications (e.g., Asatsuma-Okumura et al. Nat Chem Biol 2019 doi: 10.1038/s41589-019-0366-7) . Anti-CRBN44 was used for several publications (e.g. Lopez-Girona et al. Leukemia 2012 doi: 10.1038/leu.2012.119). All the other antibodies were validated for intended use by their respective suppliers.

Eukaryotic cell lines

Policy information about cell lines

Cell line source(s)	Lt-NES (AF22) and Sai2 were kind gifts from Prof. Austin Smith (Department of Biochemistry, Cambridge University, UK) and Prof. Hideyuki Okano (Department of Physiology, Keio University, Japan), respectively. U937 expressing PLZF-RARα (B412) was kind gifts from Prof. Estelle Duprez (Cancer Research Center of Marseille, France). 293T cells were obtained from American Type Culture Collection (USA). U937 and KG1 cells were purchased from DS pharmamedical.
Authentication	lt-NES, Sai2, and B412 cells were not authenticated. Other cell lines were authenticated by STR profiling.
Mycoplasma contamination	All cell lines were confirmed to be mycoplasma-negative using the MycoAlert Mycoplasma Detection Kit (Lonza).
Commonly misidentified lines (See ICLAC register)	No commonly misidentified cell lines were used.
